# Patient-reported outcome measures for primary hyperparathyroidism: a systematic review of measurement properties

**DOI:** 10.1186/s12955-024-02248-9

**Published:** 2024-04-02

**Authors:** George Liang, Manraj N. Kaur, Carrie G. Wade, Maria O. Edelen, David W. Bates, Andrea L. Pusic, Jason B. Liu

**Affiliations:** 1 Patient-Reported Outcomes, Value, and Experience (PROVE) Center, Department of Surgery, Brigham and Women’s Hospital, Boston, MA USA; 2 Harvard Medical School, Boston, MA USA; 3 Division of General Internal Medicine and Primary Care, Brigham and Women’s Hospital, Boston, MA USA; 4 Clinical and Quality Analysis, Information Systems, Mass General Brigham, Boston, MA USA; 5 Division of Plastic Surgery, Brigham and Women’s Hospital, Boston, MA USA; 6 Division of Surgical Oncology, Brigham and Women’s Hospital, Boston, MA USA

**Keywords:** Hyperparathyroidism, Patient reported outcome measures, Health-Related Quality of Life, Parathyroidectomy, Quality of Health Care

## Abstract

**Background:**

The quality of patient-reported outcome measures (PROMs) used to assess the outcomes of primary hyperparathyroidism (PHPT), a common endocrine disorder that can negatively affect patients’ health-related quality of life due to chronic symptoms, has not been rigorously examined. This systematic review aimed to summarize and evaluate evidence on the measurement properties of PROMs used in adult patients with PHPT, and to provide recommendations for appropriate measure selection.

**Methods:**

After PROSPERO registration (CRD42023438287), Medline, EMBASE, CINAHL Complete, Web of Science, PsycINFO, and Cochrane Trials were searched for full-text articles in English investigating PROM development, pilot studies, or evaluation of at least one PROM measurement property in adult patients with any clinical form of PHPT. Two reviewers independently identified studies for inclusion and conducted the review following the Consensus-Based Standards for the Selection of Health Measurement Instruments (COSMIN) Methodology to assess risk of bias, evaluate the quality of measurement properties, and grade the certainty of evidence.

**Results:**

From 4989 records, nine PROM development or validation studies were identified for three PROMs: the SF-36, PAS, and PHPQoL. Though the PAS demonstrated sufficient test-retest reliability and convergent validity, and the PHPQoL sufficient test-retest reliability, convergent validity, and responsiveness, the certainty of evidence was low-to-very low due to risk of bias. All three PROMs lacked sufficient evidence for content validity in patients with PHPT.

**Conclusions:**

Based upon the available evidence, the SF-36, PAS, and PHPQoL cannot currently be recommended for use in research or clinical care, raising important questions about the conclusions of studies using these PROMs. Further validation studies or the development of more relevant PROMs with strong measurement properties for this patient population are needed.

**Supplementary Information:**

The online version contains supplementary material available at 10.1186/s12955-024-02248-9.

## Introduction

Primary hyperparathyroidism (PHPT) is a common endocrine condition of parathyroid hormone over secretion due to the neoplastic overgrowth of one or multiple parathyroid glands [1]. PHPT is one of the most common causes of chronic hypercalcemia [2, 3]. Though it has an estimated prevalence of 233 cases per 100,000 women and 85 per 100,000 men, with an incidence of 66 cases per 100,000 person-years in women and 25 per 100,000 person-years in men, PHPT is underdiagnosed and underrecognized [4–6]. Left untreated, PHPT can lead to premature osteoporosis, fragility fractures, nephrolithiasis, chronic kidney disease, cardiovascular disease, and a constellation of symptoms, including fatigue, poor cognition, bone or joint pain, sleep disturbance, and anxiety, that negatively affect patients’ health-related quality of life (HRQL) [7–9]. Surgery to remove the aberrant gland(s) is currently the only curative treatment [10].

International guidelines recommend surgery only when evidence of end-organ damage is identified, such as osteoporosis on a bone density scan or when the patient passes a kidney stone [11]. The presence of symptoms and the potential to alleviate them are not considered reasons to treat patients in these guidelines because the symptoms of PHPT overlap with those of aging and are considered “vague,” “nonspecific,” and “subjective.” Though prior studies [7, 12, 13], including randomized controlled trials [14–17], have attempted to measure the symptoms of PHPT and demonstrate their improvement with surgery using patient-reported outcome (PRO) measures (PROMs), the findings have been inconsistent, resulting in considerable uncertainty about how best to care for these patients.

The controversy surrounding symptom relief and HRQL improvement in PHPT may be due to the use of PROMs with inadequate measurement properties. As with any outcome measure, selecting a rigorously developed and validated PROM is critical to accurately characterize the comparative effectiveness of treatment interventions [18, 19]. Whether a PROM is most suitable depends primarily on whether the PROM adequately measures the construct(s) of interest to the user. Therefore, we sought to systematically identify and evaluate the quality of existing PROMs used in studies of adults with PHPT by following the Consensus-Based Standards for the Selection of Health Measurement Instruments (COSMIN) Methodology [20–22]. The results of this review can help to determine which PROMs are most suitable for PHPT research and clinical practice, and outline directions for future research in this space.

## Methods

### Protocol and registration

This systematic review was conducted according to the COSMIN Methodology for Systematic Reviews of PROMs [20–22] and reported following the Preferred Reporting Items for Systematic Reviews and Meta-Analyses (PRISMA) checklist. The protocol was registered on PROSPERO (CRD42023438287) and did not require ethics approval.

### Search strategy and eligibility criteria

The databases Medline, EMBASE, CINAHL Complete, Web of Science, PsycINFO, and Cochrane Trials were systematically searched on 2 July 2023, and updated on 8 December 2023. The search strategy was developed in consultation with a clinical librarian (CW) to identify all primary research articles using any PROM in adult patients aged 18 years or older with PHPT (Additional File 1) [20]. Following the COSMIN search recommendations led to the exclusion of articles that were known *a priori* that should be included in this review. Therefore, a different search strategy was developed in consultation with our clinical librarian and clinical experts to ensure no relevant studies were missed, which included treatment strategies for primary, secondary, and tertiary hyperparathyroidism in the search to ensure high search sensitivity. No date restrictions were applied. We included any full-text articles published in English investigating PROM development, pilot studies, or evaluation of at least one PROM measurement property. At least one of the aims of the article had to be the development of a PROM or the evaluation of one or more measurement properties of a PROM for use in adults with PHPT. In articles including other conditions, patients with PHPT had to comprise 50% or more of the patients or subgroup analyses on PHPT-specific data had to be available. All forms of PHPT (i.e., classic, normocalcemic, normohormonal, hereditary, etc.) were included.

Studies that only used the PROM as an outcome measure or studies in which the PROM was used in a validation study of another instrument were excluded [20]. Articles that used PROMs but not with the intention to study the disease of PHPT were also excluded; examples of such studies include quality improvement studies (e.g., enhanced recovery after surgery, opioid minimizing perioperative pathways) and studies of surgical or anesthetic techniques. Case reports, conference abstracts, editorials, trial protocols, and theses were excluded. Review articles, consensus statements, and practice guidelines were also excluded but their bibliographies were searched to identify additional potentially eligible studies that were not identified through the database search.

### Study selection and data collection

We used Covidence (Melbourne, Victoria; Australia) to screen articles for inclusion. Two independent reviewers (GL, JBL) screened all titles and abstracts for potential full-text review. Disagreements were resolved through discussions. If a consensus could not be reached, the full-text article was retrieved. Two independent reviewers (GL, JBL) then screened full-text articles for inclusion. Disagreements at this stage were resolved by a third reviewer (MK) or discussion among the reviewers (MK, GL, JBL).

Extracted information for each article included study characteristics (author, year, country of origin, language, patient characteristics, disease characteristics, setting, response rates), PROM characteristics (construct[s] measured, target population, mode of administration, recall period, subscales, number of items, response options, scoring), and the measurement properties of the PROMs. Following the COSMIN methodology and definitions [20, 21], articles were searched for studies on (1) PROM development (2), content validity (3), structural validity (4), internal consistency (5), cross-cultural validity/measurement invariance (6), reliability (7), measurement error (8), construct validity, and (9) responsiveness. Criterion validity was not considered as there is no known “gold standard” available for measuring the construct(s) of interest in the PHPT population.

### Methodological quality and risk of bias

The methodological quality of each single study on a measurement property was extracted sequentially and assessed using the COSMIN Risk of Bias checklist by two independent reviewers (MK, JBL) [22, 23]. Each study was rated as very good, adequate, doubtful, or inadequate following the worst score counts principle. Disagreements were resolved through discussion.

The COSMIN Methodology for Assessing the Content Validity of PROMs was followed to evaluate PROM development and content validity for each PROM [21]. Existing ratings of the quality of PROM development were used when available [24–26]. Reviewer ratings were considered additional to the available evidence from the literature and were weighted less than the evidence from available development and content validity studies [21]. If there are no content validity studies, or only content validity studies of inadequate quality, and the PROM development is of inadequate quality, the rating of the reviewers will determine the overall ratings. Indirect evidence, when available, was considered for content validity only and not for other measurement properties.

Prior to evaluating structural validity, internal consistency, and cross-cultural validity/measurement invariance, each PROM’s measurement model was determined to be reflective or formative to ensure appropriate interpretations [20, 27, 28]. A “thought test” was performed to determine which model was used if one was not reported. If the PROM contained a mix of reflective and formative items, the PROM was assumed to be based on a reflective model and related measurement properties were evaluated.

In this review, a construct approach was taken to evaluate hypothesis testing for construct validity and responsiveness. Any construct known to be clinically relevant to PHPT was considered, such as fatigue, sleep disturbance, depression, anxiety, etc [7, 10, 12–17, 29]. Hypothesis testing criteria were adapted from the COSMIN manual [20]. For construct validity, these included: (1) correlation coefficients between the investigated PROM and the comparator instrument both measuring the same or similar construct(s) are 0.50 or more (2), correlation coefficients between the investigated PROM and the comparator instrument both measuring different construct(s) are 0.30 or less, and (3) effect sizes (e.g., standardized mean differences) between the scores of the investigated PROM in patients with PHPT and a different, unrelated condition are 0.8 or more. In consultation with clinical experts, patients are expected to improve three to four weeks after definitive surgical treatment (i.e., resection of the abnormal gland(s)) at least moderately. Therefore, for responsiveness, hypotheses included: (1) effect sizes of the investigated PROM are 0.30 or more, and (2) effect sizes of the investigated PROM and the comparator instrument both measuring the same or similar construct(s) are 0.30 or more.

### Evaluation of measurement properties

The results of each study on a measurement property were evaluated against the Updated Criteria for Good Measurement Properties and rated as either sufficient, insufficient, or indeterminate [20, 23]. Results from individual studies were then qualitatively summarized per measurement property per PROM. The overall result was then rated against the Updated Criteria for Good Measurement Properties to derive an overall rating of sufficient, insufficient, indeterminate, or inconsistent for the measurement property per PROM. Inconsistent results were summarized and presented separately when explanations were available. Otherwise, the conclusion was based on the majority of consistent results.

### Certainty of evidence

COSMIN’s modified Grading of Recommendations Assessment, Development, and Evaluation (GRADE) approach was used to grade the certainty of evidence considering the methodological quality of studies, total sample size, and consistency of results [20]. Specifically, the certainty of evidence was downgraded based on the risk of bias, imprecision, inconsistency, and/or indirectness, where applicable. For content validity, imprecision was not taken into account. The certainty of evidence was rated as high, moderate, low, or very low. For example, if no content validity studies were available for a PROM and PROM development was inadequate, the certainty of evidence was rated as very low. If only one study of inadequate methodological quality based on the COSMIN Risk of Bias Checklist was available, the certainty of evidence was downgraded from high to very low [20, 22]. For internal consistency, the certainty of evidence started at the level of structural validity. Following others, the certainty of the evidence was not graded for studies when the overall rating was indeterminate [23].

### Recommendations for use

Each PROM was categorized following the COSMIN methodology as: category A, recommended for use; category B, potential to be recommended for use but requires further validation; or, category C, should not be recommended for use [20]. PROMs categorized as A have evidence for sufficient content validity (any level) and at least low certainty evidence for sufficient internal consistency; results obtained from these measures are considered trustworthy. PROMs based on a formative model were categorized as A if they have evidence for sufficient content validity (any level) and at least low certainty evidence for sufficient reliability. PROMs categorized as C have high certainty evidence for an insufficient measurement property. PROMs categorized as B are those not in A or C.

## Results

### Study selection

**Fig. 1:**
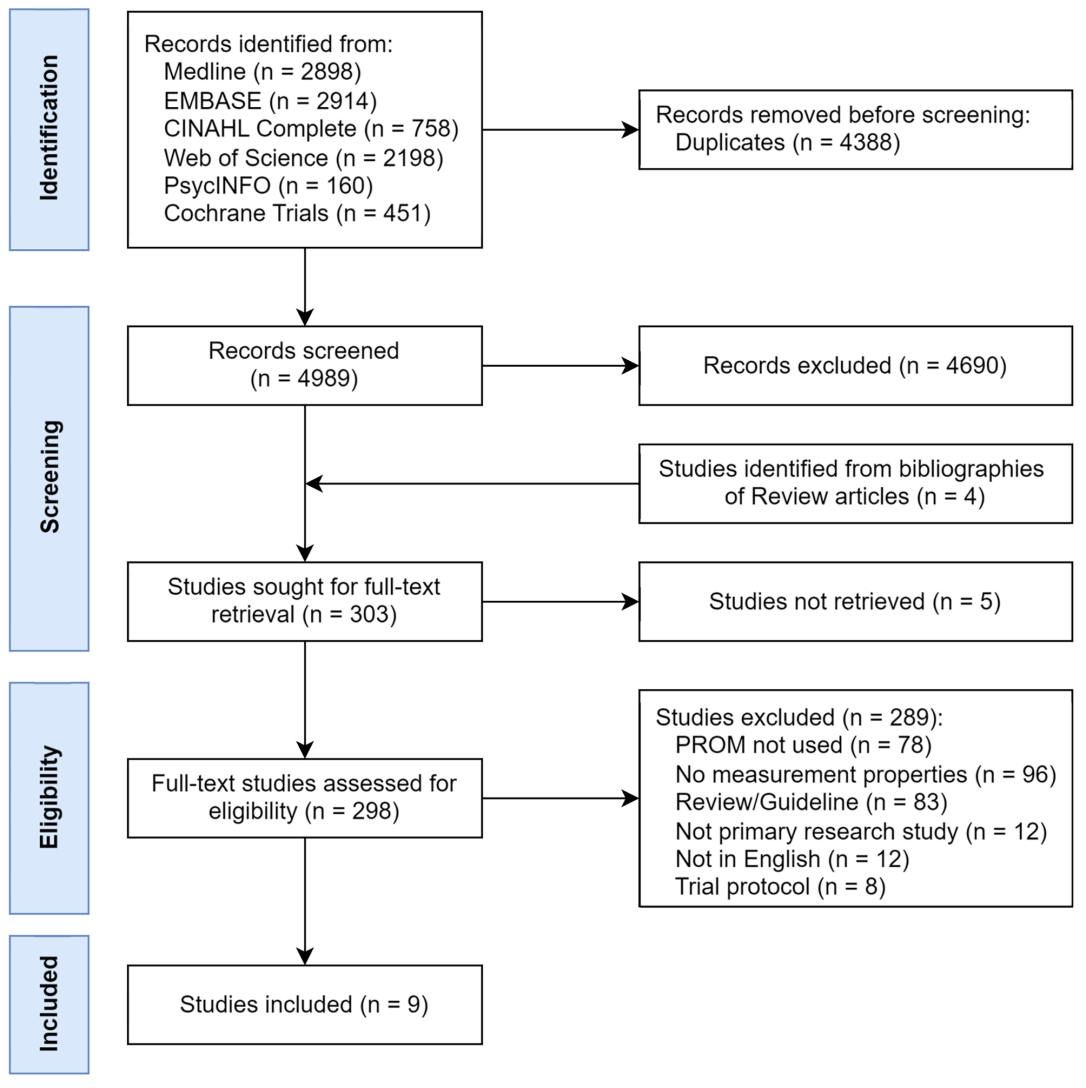
PRISMA diagram. “PROM not used” refers to articles that included potentially clinically relevant constructs, such as symptomatology or functional status, but were not evaluated using a PROM, such as ad hoc symptom checklists or neurocognitive/neuropsychological tests (e.g., Weschler Adult Intelligence Scale, Mini-Mental Status Examination, etc.). “No measurement properties” refers to articles that used PROMs (e.g., SF-36, PAS, PHPQoL, PHQ-9, GAD-7, etc.) but did not investigate their development or measurement properties

After removing duplicates, 4989 studies were identified. After screening titles and abstracts, 298 studies were retrieved for full-text review. An additional four studies were identified from searching the bibliographies of review articles and included for full-text review. There were nine studies that reported measurement properties (Fig. 1). Additional File 2 catalogues the excluded studies. Proportionate agreement and Kappa statistics among reviewers were 0.97 and 0.79, respectively, at the title and abstract screening stage, and 0.99 and 0.89, respectively, at full-text review stage.

### Study characteristics

Three PROMs with reported measurement properties were identified: one generic, the 36-item Short Form Survey (SF-36), and two PHPT-specific, the Parathyroidectomy Assessment of Symptoms (PAS) and the Primary Hyperparathyroidism Quality of Life (PHPQoL) measure. Tables 1 and 2 contain an overview of the identified PROMs and a description of the study populations, respectively. All three PROMs are self-administered multi-item instruments.

**Table 1:** Characteristics of included patient-reported outcome measures (PROMs).

PROM	Construct	Target Population	Mode of Administration	Recall Period	Total Number of Items	Subscales (Number of Items)	Response Options	Range of Scores/Scoring
SF-36	Quality of life	PHPT	Self-administered	Past 4 weeks	36	Physical functioning (10), role limitations-physical (4), role limitations-emotional (3), energy (4), emotional well-being (5), social functioning (2), pain (2), general health (5)	Variable adjectival scales	0-100 (higher scores indicate better quality of life)
PAS	Symptom presence and severity	PHPT	Self-administered	Today	13	Bone pain (1), feeling tired easily (1), mood swings (1), feeling ‘‘blue’’ or depressed (1), pain in the abdomen (1), feeling weak (1), feeling irritable (1), pain in the joints (1), being forgetful (1), difficulty getting out of a chair or car (1), headaches (1), itchy skin (1), being thirsty (1)	0-100 visual analogue scales (0 is none, 100 is extreme)	0-1300 (lower scores indicate lower symptom burden)
PHPQoL	Quality of life	PHPT	Self-administered	Past 4 weeks	16	Physical functioning (9), emotional functioning (7)	5-point Likert adjectival scales (always, many times, from time to time, hardly ever, and never) except 1 item that includes an additional “not applicable” response option	0-100 (higher scores indicate better quality of life)

SF-36: 36-item Short Form Survey; PAS: Parathyroidectomy Assessment of Symptoms; PHPQoL: Primary Hyperparathyroidism Quality of Life

PHPT: primary hyperparathyroidism

**Table 2:** Characteristics of the included studies

PROM	Reference	Sample Size	Age, y	Female, %	Clinical Details	Setting	Country	Language	Response Rate
SF-36	Burney 1996	59	Mean 59.9 (range 30–86)	77%	PHPT	Outpatient	USA	English	Baseline: NR 2 months: 67.8% 6 months: 39.0%
	Burney 1998	140	Mean 58 (range 21–85)	74%	PHPT	Outpatient	USA	English	Baseline: NR 2 months: 78.6% 6 months: 48.5%
	Burney 1999	155	Low calcium group: mean 57 High calcium group: mean 59	Low calcium group: 71% High calcium group: 74%	PHPT; considered two groups above (high calcium) and below (low calcium) 10.9 mg/dL	Outpatient	USA	English	Baseline: NR 2 months: NR 6 months: 67.1%
PAS	Pasieka 1998	63	Mean 54 (range 13–80)	75%	PHPT vs. thyroid disease (*n* = 54)	Outpatient	Canada	English	Baseline: NR 1 week: NR 3 months: NR 12 months: NR
	Pasieka 2002	203 (Australia: 27, USA: 54, Canada: 122)	Australia: mean 52 (range 15–76) USA: mean 52 (range 25–77) Canada: mean 55 (range 13–81)	Australia: 100% USA: 65% Canada: 73%	PHPT vs. thyroid disease (*n* = 58 from Canada)	Outpatient	Australia, USA, Canada	English	Australia Baseline: 100% 1 week: 100% 3 months: 100% 12 months: 0% USA Baseline: 100% 1 week: 100% 3 months: 100% 12 months: 100% Canada Baseline: NR 1 week: NR 3 months: NR 12 months: NR
	Mihai and Sadler 2008	101	Mean 61 (SD 17; range 18–89 years)	70.3%	PHPT	Outpatient	UK	English	Baseline: 100% 3 months: 73.3% 6 months: 48.5% 12 months: 67.3%
	Tzikos 2022	50	Mean 64 (SD 12.7)	NR	PHPT	Outpatient	Greece	Greek	Baseline: 100% 1 month: 100%
PHPQoL	Webb 2013	67	Mean 59.2 (SD 13.4)	69.7%	PHPT	Outpatient	Spain	Spanish	80.9%
	Webb 2016	182	Mean 61.4 (SD 12.1)	79.7%	PHPT	Outpatient	Spain	Spanish	NR

NR: not reported; SD: standard deviation

SF-36: 36-item Short Form Survey; PAS: Parathyroidectomy Assessment of Symptoms; PHPQoL: Primary Hyperparathyroidism Quality of Life

PHPT: primary hyperparathyroidism

### SF-36

Three articles reported the internal consistency of the SF-36 in adult patients with PHPT (Additional File 3) [30–32]. The methodological quality of the studies using the COSMIN Risk of Bias checklist was each doubtful as the risk of bias in a study on internal consistency depends on the available evidence for structural validity because unidimensionality is a prerequisite for the interpretation of internal consistency analyses (e.g., Cronbach’s alpha) [20, 22]. Therefore, the certainty of evidence for internal consistency cannot be higher than the certainty of evidence for structural validity. As no studies on the structural validity of the SF-36 in this patient population were identified, the certainty of evidence for the reported internal consistency analyses could not be graded (Table 3). That is, internal consistency was rated as indeterminate against the Updated Criteria for Good Measurement Properties despite Cronbach’s alpha values greater than 0.8 for each subscale. No other measurement properties for the SF-36 in the target population were found, including content validity. The SF-36 development was previously evaluated and determined to be inadequate [21]. Considering indirect evidence and the reviewers’ ratings, there was very low certainty evidence of sufficient content validity [24, 26].

**Table 3:** Summary of findings using COSMIN’s modified GRADE criteria

			SF-36	PAS	PHPQoL
**PROM Development**		*Design*	Inadequate	NR	Inadequate
		*Pilot Study*	NR	NR	NR
		*Overall*	Inadequate	N/A	Inadequate
**Content Validity**	**Relevance**	*Rating*	Sufficient	Sufficient	Insufficient
		*GRADE*	Very Low	Very Low	Very Low
	**Comprehensiveness**	*Rating*	Insufficient	Sufficient	Insufficient
		*GRADE*	Very Low	Very Low	Very Low
	**Comprehensibility**	*Rating*	Sufficient	Insufficient	Insufficient
		*GRADE*	Very Low	Very Low	Very Low
**Measurement Properties**	**Structural Validity**	*Rating*	NR	N/A	Indeterminate
		*GRADE*	NR	N/A	N/A
	**Internal Consistency**	*Rating*	Indeterminate	N/A	Indeterminate
		*GRADE*	N/A	N/A	N/A
	**Cross-cultural Validity /** **Measurement Invariance**	*Rating*	NR	N/A	NR
		*GRADE*	NR	N/A	NR
	**Reliability**	*Rating*	NR	Sufficient	Sufficient
		*GRADE*	NR	Very Low	Very Low
	**Construct Validity**	**Convergent Validity**			
		*Rating*	NR	Sufficient	Sufficient
		*GRADE*	NR	Very Low	Very Low
		**Discriminative Validity**			
		*Rating*	NR	Insufficient	Indeterminate
		*GRADE*	NR	Low	N/A
	**Responsiveness**	*Rating*	NR	Insufficient	Sufficient
		*GRADE*	NR	Low	Very Low

No studies reported measurement error. Criterion validity was not evaluated as there is no measurement “gold standard.”

NR: not reported. N/A: not applicable

GRADE: Grading of Recommendations Assessment, Development, and Evaluation; SF-36: 36-item Short Form Survey; PAS: Parathyroidectomy Assessment of Symptoms; PHPQoL: Primary Hyperparathyroidism Quality of Life

### PAS

No PROM development or content validity studies were identified for the PAS. Because no content validity studies were found, solely the reviewers’ ratings counted for the evidence synthesis, leading to very low certainty evidence of sufficient content validity (Table 3). Internal consistency and test-retest reliability of the PAS were mentioned in one article [33], but the source study for these measurement properties was not specified and could not be located. Using the “thought test,” the PAS was based on a formative model and thus structural validity, internal consistency, and cross-cultural validity/measurement invariance were not applicable, thus studies reporting these measurement properties were ignored [27, 28, 34]. Test-retest reliability was evaluated in a Greek translation study, which was rated as sufficient but with very low certainty of evidence due to risk of bias and imprecision (Table 3; Additional File 4) [35].

Three articles reported on construct validity, specifically convergent and discriminative validity, of the PAS [33, 36, 37]. One article examined the convergent validity of the PAS with the SF-36 [37]. Correlation coefficients satisfied our hypothesis for construct validity and thus convergent construct validity was rated as sufficient against the Updated Criteria for Good Measurement Properties. However, the methodologic quality was inadequate because the SF-36, following the COSMIN methodology [20–22], does not have high-quality measurement properties in this patient population, precluding interpretation of these correlation coefficients, downgrading the certainty of evidence. The other two articles examined discriminative validity of the PAS by comparing PAS scores to those from a cohort of unrelated patients with thyroid disease [33, 36]. Statistical significance rather than effect sizes was reported, therefore discriminative validity was rated insufficient. The certainty of evidence was very low and low, respectively, for convergent validity and discriminative validity due to the risk of bias.

Responsiveness of the PAS was assessed in two studies [33, 36]. Neither study hypothesized the expected magnitude of the effect, defined a clinically relevant time interval, or calculated effect size estimates. However, results showed statistically significant score improvement after surgery as clinically expected. The methodological quality of the studies was each inadequate, and responsiveness was rated overall as insufficient since only statistical significance was evaluated. Significant change is not equivalent to valid change, thus precluding our ability to apply our criteria for hypothesis testing for responsiveness [20]. The overall certainty of evidence was low due to risk of bias.

### PHPQoL

Two articles reported PHPQoL development and its measurement properties (Table 3; Additional Files 5–6) [38, 39]. Though the construct, conceptual framework, and intended use for the PHPQoL were clearly delineated, no concept elicitation study was conducted with patients to identify important domains and to generate items. Instead, experts identified the most important domains and qualitative interviews with 24 patients were conducted to develop items within the expert-defined domains. No patients were subsequently involved in item selection based on relevance, comprehensiveness, and comprehensibility. No cognitive debriefing interview studies were conducted to demonstrate content validity, and thus development of the PHPQoL was rated as inadequate. The relevance, comprehensiveness, and comprehensibility were all rated as insufficient, resulting in the content validity of the PHPQoL to be rated insufficient [21]. The certainty of evidence was judged to be very low because no content validity studies were available and PHPQoL development was inadequate.

Structural validity, internal consistency, reliability, construct validity, and responsiveness of the PHPQoL were reported. Cross-cultural validity of an English translation of the PHPQoL was mentioned but results not reported, and thus could not be rated. Structural validity was rated as indeterminate because although exploratory factor analysis was conducted, no model fit statistics were reported, thus not meeting the Criteria for Good Measurement Properties [23]. As the PHPQoL was developed using a reflective model, the reported Cronbach’s alpha coefficients would have been judged to be sufficient. However, the risk of bias in a study on internal consistency depends on the available evidence for structural validity [20]. Therefore, internal consistency was also rated as indeterminate. Test-retest reliability was conducted in 78 patients with an ICC > 0.8 and rated as sufficient. The certainty of evidence was very low due to risk of bias and imprecision.

Hypothesis testing for construct validity, specifically discriminative validity, was reported in the development paper. However, the hypotheses tested in the development paper were different than the ones specified in this review, resulting in an indeterminate rating. Statistical significance rather than effect sizes was reported yielding inadequate methodologic quality. Convergent validity was sufficient based on results of hypothesis testing in the validation study that satisfied our defined criterion with correlation coefficients greater than 0.5. However, the methodologic quality was inadequate because neither the SF-36 nor the Psychological Well-Being Index (PWBI) have demonstrated high-quality measurement properties in this patient population for appropriate comparison to the PHPQoL, downgrading the certainty of evidence [20, 21]. Responsiveness of the PHPQoL was rated as sufficient having met our defined criteria. In summary, COSMIN’s modified GRADE approach grade for convergent validity and responsiveness were both very low due to risk of bias. Discriminative validity was not graded as it was rated indeterminate.

### Recommendations for use

All PROMs were categorized as B (Table 3). None had evidence for sufficient content validity of any level and at least low certainty evidence for sufficient internal consistency (or reliability for the PAS), nor high certainty evidence for an insufficient measurement property.

## Discussion

Though PHPT is recognized to cause symptoms that can negatively affect HRQL, debate continues as to whether these aspects of the disease can be measured or remedied with treatment [10, 11]. This controversy might stem in part from the use of PROMs in research studies with poor measurement properties or those irrelevant for this patient population, resulting in inconsistent findings. This systematic review provides a synthesized methodological evaluation of the measurement properties of PROMs used in adult patients with PHPT following the COSMIN methodology [20–22]. Nine studies reported on the measurement properties of three PROMs: the SF-36, the PAS, and the PHPQoL. Based on the COSMIN methodology, none can be currently recommended for use in clinical practice or research studies to detect PHPT or evaluate treatment effectiveness due to limited content validity, conceptual weaknesses, methodological shortcomings, and/or low certainty evidence, though they are useful for other purposes. These results raise important questions about the conclusions of studies using these PROMs in adults with PHPT and underscore the need for further validation studies or the development of more relevant PROMs for this patient population.

The SF-36 is by far the most used PROM to assess PROs in patients with PHPT [7, 12], and was used as the primary endpoint in a randomized controlled trial that forms the empirical basis of current international clinical guidelines [16]. However, based on the COSMIN Methodology [20, 21], the findings of this review suggest that the SF-36 cannot currently be recommended for use in research or clinical care in patients with PHPT because the content validity of the SF-36 was not established in patients with PHPT, and the only measurement property that could be evaluated was internal consistency (i.e., category B). Yet, the SF-36 carries considerable validity and reliability as a universal PROM with global and domain-specific scales for patients with chronic conditions. It is widely used and accepted to assess general HRQL across varied patient populations [24, 26]. The SF-36 measures several domains hypothesized to be clinically relevant in PHPT, including vitality and social functioning, and thus its use is ostensibly appropriate. However, according to the COSMIN Methodology [21], “researchers do not validate the PROM, but rather the application of the PROM;” thus, measurement properties should be established in the target population. Although we considered indirect evidence for its content validity, further studies demonstrating high certainty evidence for the SF-36 in patients with PHPT are needed before the SF-36 can be recommended for use. As the PAS and the PHPQoL demonstrate, other relevant domains and condition-specific concerns are important to measure for patients with PHPT, and less so for others, suggesting that the SF-36 may not be relevant or comprehensive for patients with PHPT. This is not surprising as the SF-36 is a universal PROM.

When considering universal PROMs that are applied to a narrow population, like the SF-36 in patients with PHPT, the COSMIN Methodology could be viewed as overly strict. Universal PROMs are designed to measure outcomes from patients with a broad range of conditions and health statuses. Patients in a range of target populations may have been included in PROM development and content validity studies, thus providing indirect evidence for content validity. The COSMIN Methodology does acknowledge consideration of indirect evidence when evaluating content validity [21]. The SF-36 has good content validity in the original diverse target population, but it is unknown whether patients with PHPT, or how many, were included in the original development and content validity studies of the SF-36, thus downgrading the certainty of evidence for indirectness.

Another key tenet of choosing a PROM is its intended use. As a universal PROM, the SF-36 is useful to compare groups with diverse conditions, including PHPT, and less suited to detect change to treatment among only patients with PHPT [40–42]. Therefore, if the intent of the PROM is to demonstrate treatment effectiveness, a domain- or condition-specific PROM, like the PAS or the PHPQoL, may be more responsive to change (though not always the case). From the perspective of “intended use,” evaluating the measurement properties of the SF-36 could be considered unnecessary since the SF-36, as a universal PROM, may not be the most appropriate PROM to detect changes due to treatment. Yet still, modern PROMs using advanced psychometrics that are applicable to diverse patient populations can demonstrate clinically relevant responsiveness and could be considered in future research [43, 44].

The PHPQoL had the strongest content validity because a clear conceptual framework was established, and patients were involved in item generation. However, no concept elicitation studies, cognitive interviews, or content validity studies involving patients were identified. Validation in English was not reported and thus could not be assessed. These shortcomings combined with the limited evidence on the other measurement properties resulted in our assessment of a category B rating for use. Nevertheless, the development and validation of the PHPQoL involved considerable psychometric expertise and analyses, which may not have met COSMIN criteria due to reporting bias. Additional studies to address these shortcomings could easily improve the PHPQoL rating for use to category A.

We identified two flaws of the PAS. First, we could not identify any development or content validity studies. Content validity is a critically important measurement property– it requires that the items of the PROM are relevant, comprehensive to the construct(s), and comprehensible to the population of interest, thus ensuring the PROM is measuring what it is intended to measure [20–22]. PROM development was reported in a conference abstract, but this could not be retrieved, nor could it be included in our review as it was not a full-text study. Hence, it is unclear whether patients were involved in the development of the PAS and whether it reflects the concepts that matter to them. Second, the PAS was constructed using a formative measurement model [27, 28, 34]. Formative models apply to constructs that are represented by different domains or components, so that constructs in formative models are not unidimensional, but rather result from the combination of heterogeneous indicators. Items in a scale or subscale based on a formative model are not supposed to be correlated and the evaluation of the internal structure of such PROMs is not applicable. Thus, the structural validity, internal consistency, and cross-cultural validity/measurement invariance could not be evaluated for the PAS [20]. Though no high certainty evidence for insufficient measurement properties could be identified for the PAS to be deemed unsuitable for use (i.e., category C) following the COSMIN methodology, the PAS did not have evidence for sufficient content validity of any level and at least low certainty evidence for sufficient reliability, thus resulting in a category B designation [21]. Given the clinical uptake of the PAS and its arguable utility as a clinical index [12, 45], an updated version following COSMIN methodology to ensure strong measurement properties with appropriate scoring based on its formative model may prove fruitful.

Numerous studies, including randomized clinical trials [7, 12, 14–17], over the last 30 + years have attempted to demonstrate the effectiveness of surgery to alleviate the symptoms of PHPT and improve HRQL. However, these studies continue to report mixed results, thus preventing any clinical practice guidelines from recommending surgery for symptom and HRQL improvement [11]. We believe that the inconsistent results from this large body of research may stem from the use of inadequate PROMs with poor measurement properties to measure symptoms and their impact on HRQL in patients with PHPT. The specification of an outcome in research is vital to ensure the accuracy of its findings. By using an inadequate PROM, study results are fundamentally flawed. Future research into this realm of PHPT would benefit from additional validation studies on the identified PROMs or the development of a more relevant PROM, or set of PROMs, with strong measurement properties.

Two other potential explanations for the failure to detect the effectiveness of surgery are possible and may coexist with poor PROM measurement properties. When PROMs are used as the primary outcome in a clinical trial, the identification and quantification of subtle changes due to treatment are critical since the success or failure of the trial depends entirely on the PROM. It is therefore essential that the PROM be responsive to small, but important, changes to determine if the treatment is effective or potentially harmful [46]. As discussed earlier, universal (a.k.a. generic) measures, like the SF-36, are less likely to be responsive to clinical interventions compared to condition-specific measures, particularly at the individual level [40–42]. Future studies attempting to determine the effectiveness of surgery in PHPT should include condition-specific measures in addition to universal ones. Modern PROMs based on item response theory may serve dual purpose [44].

The second explanation is response shift [47, 48], which refers to a change in the meaning of one’s self-evaluation because of changes in internal standards (recalibration), values (reprioritization), and/or conceptualization of the target construct (reconceptualization). After surgery, patients with PHPT may experience short-term improvements in their symptoms and HRQL that become their “new normal,” blunting the ability to detect changes over time. Response shift can lead to the erroneous conclusion that surgery provides no long-term benefit to a patient’s HRQL when the opposite may be true.

This study has several limitations. Though we searched six databases and the references of review articles using a very broad and sensitive search strategy, it is possible important development and validation studies were missed. Furthermore, the inability to retrieve some potential studies, the exclusion of studies in languages other than English, or the omission of measurement properties in published studies limited the number of included studies. We attempted to mitigate reviewer bias by using two independent reviewers at all stages of the review process. However, subjectivity in our ratings remains due to certain aspects of the COSMIN methodology [20–22]. Last, indirect evidence was considered for content validity only and not for other measurement properties. This may have limited our ability to rate certain measurement properties, but the certainty of evidence would have been downgraded regardless for indirectness.

## Conclusions

This systematic review, conducted with the COSMIN methodology, identified three PROMs (i.e., SF-36, PAS, PHPQoL) with little-to-no content validity and insufficient measurement properties based on low-certainty evidence for this patient population. Until high-quality validation studies become available, the conclusions of studies using these three PROMs, regardless of whether they support or refute the effectiveness of surgery, may be flawed. Furthermore, any conclusions drawn from studies using other PROMs without any evaluation of their measurement properties in this patient population are even more suspect. The development of more relevant PROMs with strong measurement properties following the COSMIN methodology could also be considered to improve detection and treatment of PHPT, which could in turn improve the quality of care for patients with PHPT.

### Electronic supplementary material

Below is the link to the electronic supplementary material.

Supplementary Material 1

Supplementary Material 2

Supplementary Material 3

Supplementary Material 4

Supplementary Material 5

Supplementary Material 6

## Data Availability

No datasets were generated or analysed during the current study.

## Abbreviations

COSMIN: Consensus-Based Standards for the Selection of Health Measurement Instruments
HRQL: Health-related quality of life
PAS: Parathyroidectomy Assessment of Symptoms measure
PHPQoL: Primary Hyperparathyroidism Quality of Life measure
PHPT: Primary hyperparathyroidism
PRO: Patient-reported outcome
PROM: Patient-reported outcome measure
SF-36: Short Form Survey 36-item measure
