# Supplementary material for: Patient-reported outcome measures for primary hyperparathyroidism: a systematic review of measurement properties
Source: Health Qual Life Outcomes. 2024 Apr 2;22:31. doi: 10.1186/s12955-024-02248-9 (PMC10988805; doi:10.1186/s12955-024-02248-9)
Supplement: Supplementary file 2 — Supplementary Material 2 [file 12955_2024_2248_MOESM2_ESM.docx]

Additional File 4. Results of studies on measurement properties for the PAS.

| **PROM (ref)** | **Country (language) in which the PROM was evaluated** | **Structural validity** | | | **Internal consistency** | | | | **Cross-cultural validity\measurement invariance** | | | | | **Reliability** | | |
| --- | --- | --- | --- | --- | --- | --- | --- | --- | --- | --- | --- | --- | --- | --- | --- | --- |
|  |  | n | Meth qual | Result (rating) | n | Meth qual | | Result (rating) | n | Meth qual | | Result (rating) | | n | Meth qual | Result (rating) |
| PAS (Pasieka 1998) | Canada (English) |  |  |  |  |  | |  |  |  | |  | |  |  |  |
| PAS (Pasieka 2002) | Canada, US, Australia (English) |  |  |  |  |  | |  |  |  | |  | |  |  |  |
| PAS (Mihai and Sadler 2008) | UK (English) |  |  |  |  |  | |  |  |  | |  | |  |  |  |
| PAS (Tzikos 2022) | Greece (Greek) |  |  |  | 50 | N/A | | N/A | 50 | N/A | | N/A | | 50 | Doubtful | ICCs per item 0.850-0.968 (+) |
| **Pooled or summary result (overall rating)** | |  |  |  | **50** |  | | **N/A** | **50** |  | | **N/A** | | **50** |  | **(+)** |
| **PROM** | **Country (language) in which the PROM was evaluated** | **Measurement error** | | | **Criterion validity** | | | **Hypotheses testing** | | | | | **Responsiveness** | | | |
|  |  | n | Meth qual | Result (rating) | n | Meth qual | Result (rating) | n | Meth qual | | Result (rating) | | n | | Meth qual | Result (rating) |
| PAS (Pasieka 1998) | Canada (English) |  |  |  |  |  |  | 63 | Inadequate | | Statistically significantly higher scores in PHPT patients compared to thyroid patients (p<0.001) (-) | | 63 | | Inadequate | Statistically significant difference between baseline and 1 week postoperative (<0.001) (-) |
| PAS (Pasieka 2002) | Canada, US, Australia (English) |  |  |  |  |  |  | 122 | Inadequate | | Statistically significantly higher scores in PHPT patients compared to thyroid patients (p<0.01) (-) | | Australia: 27  USA: 54  Canada: 122 | | Inadequate | Statistically significant difference between baseline and postoperative timepoints across all three Centers (p<0.001) (-) |
| PAS (Mihai and Sadler 2008) | UK (English) |  |  |  |  |  |  | 101 | Inadequate | | SF-36 (PCS): correlation coefficient 0.55 (+)  SF-36 (MCS): correlation coefficient 0.61 (+) | |  | |  |  |
| PAS (Tzikos 2022) | Greece (Greek) |  |  |  |  |  |  |  |  | |  | |  | |  |  |
| **Pooled or summary result (overall rating)** | |  |  |  |  |  |  | **286** |  | | **(±)*** | | **266** | |  | **(-)** |

N/A: not applicable as PROM based on a formative model

* Inconsistent results were reported separately as convergent validity and discriminative validity.
