# Supplementary material for: Patient-reported outcome measures for primary hyperparathyroidism: a systematic review of measurement properties
Source: Health Qual Life Outcomes. 2024 Apr 2;22:31. doi: 10.1186/s12955-024-02248-9 (PMC10988805; doi:10.1186/s12955-024-02248-9)
Supplement: Supplementary file 3 — Supplementary Material 3 [file 12955_2024_2248_MOESM3_ESM.docx]

Additional File 5. Results of studies on measurement properties for the PHPQoL.

| **PROM (ref)** | **Country (language) in which the PROM was evaluated** | **Structural validity** | | | **Internal consistency** | | | | **Cross-cultural validity/**  **measurement invariance** | | | | | **Reliability** | | |
| --- | --- | --- | --- | --- | --- | --- | --- | --- | --- | --- | --- | --- | --- | --- | --- | --- |
|  |  | n | Meth qual | Result (rating) | n | Meth qual | | Result (rating) | n | Meth qual | | Result (rating) | | n | Meth qual | Result (rating) |
| PHPQoL (Webb 2013) | Spain (Spanish) | 67 | Inadequate | EFA: 2 domains (51.5% total variance; factor 1: 44.5%, factor 2: 7%) with factor loadings of each item on its factor ≥0.4; 1 item on both factors; (+)  Rasch: Infit 0.62-1.26; Outfit 0.65-1.29 (?) | 67 | Very good | | Cronbach’s alpha 0.82, 0.91, 0.92 (+) | NR | Doubtful | | NR (?) | |  |  |  |
| PHPQoL (Webb 2016) | Spain (Spanish) | 182* | Adequate | EFA: scree plot in line with construct; Varimax rotation (?) | 182* | Very good | | Cronbach’s alpha >0.8 (+) |  |  | |  | | 78* | Doubtful | ICC >0.8 (+) |
| **Pooled or summary result (overall rating)** | | **249** |  | **(?)** | **249** |  | | **(?)†** | **NR** |  | | **(?)** | | **78** |  | **(+)** |
| **PROM** | **Country (language) in which the PROM was evaluated** | **Measurement error** | | | **Criterion validity** | | | **Hypotheses testing** | | | | | **Responsiveness** | | | |
|  |  | n | Meth qual | Result (rating) | n | Meth qual | Result (rating) | n | Meth qual | | Result (rating) | | n | | Meth qual | Result (rating) |
| PHPQoL (Webb 2013) | Spain (Spanish) |  |  |  |  |  |  | 67 | Inadequate | | Lower scores in women than men (*p*<0.01) (?)  Worse scores with increasing age (*p*=0.52) (?)  No differences between those who did and did not have surgery (*p*=0.074) (?)  No differences between those who did and did not have kidney stones (*p*=0.23) (?) | |  | |  |  |
| PHPQoL (Webb 2016) | Spain (Spanish) |  |  |  |  |  |  | 170-176 | Inadequate | | PWBI: correlation coefficients 0.695-0.852 (+)  SF-36: correlation coefficients 0.626-0.778 (+) | | 91* | | Inadequate | Effect sizes at 3, 6, and 12 months after treatment 0.37, 0.48, 0.46 (+) |
| **Pooled or summary result (overall rating)** | |  |  |  |  |  |  | **237-243** |  | | **(±)** | | **91** | |  | **(+)** |

* Exact sample size not reported; † rated indeterminate as structural validity was rated indeterminate; NR: not reported
