# Supplementary material for: Patient-reported outcome measures for primary hyperparathyroidism: a systematic review of measurement properties
Source: Health Qual Life Outcomes. 2024 Apr 2;22:31. doi: 10.1186/s12955-024-02248-9 (PMC10988805; doi:10.1186/s12955-024-02248-9)
Supplement: Supplementary file 4 — Supplementary Material 4 [file 12955_2024_2248_MOESM4_ESM.docx]

Additional File 4. Results of studies on content validity for the PHPQoL.

| **Name of PROM**: Primary Hyperparathyroidism Quality of Life (PHPQoL) | | **PROM Development Study** | **Rating of Reviewers** | **Overall Ratings per PROM** | **GRADE Quality of Evidence** |
| --- | --- | --- | --- | --- | --- |
| Criteria | | + / - / ± / ? | + / - / ± / ? | + / - / ± | High, Moderate, Low, Very Low |
| **Relevance** | |  |  |  |  |
| 1 | Are the included items relevant for the construct of interest? | - | ? |  |  |
| 2 | Are the included items relevant for the target population of interest? | - | ? |  |  |
| 3 | Are the included items relevant for the context of use of interest? | + | ? |  |  |
| 4 | Are the response options appropriate? | - | ? |  |  |
| 5 | Is the recall period appropriate? | - | - |  |  |
| **RELEVANCE RATING** | | - | ? | - | Very Low |
| **Comprehensiveness** | |  |  |  |  |
| 6 | Are all key concepts included? | - | ? |  |  |
| **COMPREHENSIVENESS RATING** | | - | ? | - | Very Low |
| **Comprehensibility** | |  |  |  |  |
| 7 | Are the PROM instructions understood by the population of interest as intended? | - |  |  |  |
| 8 | Are the PROM items and response options understood by the population of interest as intended? | - |  |  |  |
| 9 | Are the PROM items appropriately worded? |  | - |  |  |
| 10 | Do the response options match the question? |  | + |  |  |
| **COMPREHENSIBILITY RATING** | | - | +/- | - | Very Low |
| **CONTENT VALIDITY RATING** | | - | ? | - | Very Low |
