# Supplementary material for: Patient-reported outcome measures for primary hyperparathyroidism: a systematic review of measurement properties
Source: Health Qual Life Outcomes. 2024 Apr 2;22:31. doi: 10.1186/s12955-024-02248-9 (PMC10988805; doi:10.1186/s12955-024-02248-9)
Supplement: Supplementary file 5 — Supplementary Material 5 [file 12955_2024_2248_MOESM5_ESM.pdf]

	Authors	Published Year	Title	Journal	Reason for Exclusion
1	Aberg, Viveca; Norenstedt, Sophie; Zedenius, Jan; Saaf, Maria; Nordenstrom, Jorgen; Pernow, Ylva; Nilsson, Inga-Lena	2015	Health-related quality of life after successful surgery for primary hyperparathyroidism: no additive effect from vitamin D supplementation: results of a double-blind randomized study	European journal of endocrinology	No measurement properties
2	Adkisson, Cameron D.; Yip, Linwah; Armstrong, Michaele J.; Stang, Michael T.; Carty, Sally E.; McCoy, Kelly L.	2014	Fibromyalgia symptoms and medication requirements respond to parathyroidectomy	Surgery	No measurement properties
3	Adler, J. T.; Sippel, R. S.; Schaefer, S.; Chen, H.	2009	Surgery improves quality of life in patients with "mild" hyperparathyroidism	American journal of surgery	No measurement properties
4	Adler, Joel T.; Sippel, Rebecca S.; Chen, Herbert	2008	The Influence of Surgical Approach on Quality of Life After Parathyroid Surgery	Ann Surg Oncol	No measurement properties
5	Akerstrom, G.; Ljunghall, S.	1993	Medical and radiologic evaluation and operative treatment of primary hyperparathyroidism	Current opinion in general surgery	Review/Guideline
6	Alex, Gillian; Morris, Lilah; Pasieka, Janice; Perrier, Nancy	2013	Nonclassical symptoms of primary hyperparathyroidism and their response to parathyroidectomy	The American surgeon	Review/Guideline
7	Allerheiligen, D. A.; Schoeber, J.; Houston, R. E.; Mohl, V. K.; Wildman, K. M.	1998	Hyperparathyroidism	American family physician	Review/Guideline
8	Alveryd, A.; Bostrom, H.; Wengle, B.; Wester, P. O.	1976	Indications for surgery in the elderly patient with primary hyperparathyroidism	Acta chirurgica Scandinavica	PROM not used
9	Ambrogini, Elena; Cetani, Filomena; Cianferotti, Luisella; Vignali, Edda; Banti, Chiara; Viccica, Giuseppe; Oppo, Annalisa; Miccoli, Paolo; Berti, Piero; Bilezikian, John P.; Pinchera, Aldo; Marcocci, Claudio	2007	Surgery or surveillance for mild asymptomatic primary hyperparathyroidism: a prospective, randomized clinical trial	The Journal of clinical endocrinology and metabolism	No measurement properties
10	Amin, Amanda; Wang, Tracy; Wade, Thomas; Yen, Tina	2011	Normal PTH Levels in Primary Hyperparathyroidism: Still the Same Disease?	Annals of surgical oncology	PROM not used

11	Amstrup, Anne Kristine; Rejnmark, Lars; Mosekilde, Leif	2011	Patients with surgically cured primary hyperparathyroidism have a reduced quality of life compared with population-based healthy sex-, age-, and season-matched controls	European journal of endocrinology	No measurement properties
12	Anagnostis, P.; Vaitsi, K.; Veneti, S.; Potoupni, V.; Kenanidis, E.; Tsiridis, E.; Papavramidis, T. S.; Goulis, D. G.	2021	Efficacy of parathyroidectomy compared with active surveillance in patients with mild asymptomatic primary hyperparathyroidism: a systematic review and meta-analysis of randomized-controlled studies	Journal of endocrinological investigation	Review/Guideline
13	Anastasilakis, Dimitrios A.; Makras, Polyzois; Polyzos, Stergios A.; Anastasilakis, Athanasios D.; Part of the, Combo Endo Team	2019	Asymptomatic and normocalcemic hyperparathyroidism, the silent attack: a combo-endocrinology overview	Hormones (Athens, Greece)	Review/Guideline
14	Anderson, J.	1968	The psychiatric aspects of disturbed calcium metabolism. Psychiatric aspects of primary hyperparathyroidism	Proceedings of the Royal Society of Medicine	Not primary research study
15	Anonymous,	1990	Diagnosis and management of asymptomatic primary hyperparathyroidism. National Institutes of Health Consensus Development Conference. October 29-31, 1990	Consensus statement. National Institutes of Health Consensus Development Conference	Review/Guideline
16	Anonymous,	2010	Managing primary hyperparathyroidism in primary care	Drug and therapeutics bulletin	Review/Guideline
17	Anonymous,	1991	NIH conference. Diagnosis and management of asymptomatic primary hyperparathyroidism: consensus development conference statement	Annals of internal medicine	Review/Guideline
18	Applewhite, Megan K.; Schneider, David F.	2014	Mild primary hyperparathyroidism: a literature review	The oncologist	Review/Guideline
19	Asagoe, T.; Takami, H.; Hanatani, Y.; Kodaira, S.	1995	[Parathyroid crisis]	Nihon rinsho. Japanese journal of clinical medicine	Not English
20	Ayuk, John; Cooper, Mark S.; Gittoes, Neil J. L.	2010	New perspectives in the management of primary hyperparathyroidism	Therapeutic advances in endocrinology and metabolism	Review/Guideline
21	Ã–zoÇ§ul, B.; Kisaoglu, A.; AkÃ§ay, M. N.; Atamanalp, S. S.; Yildirgan, M. I.; Ã–ztÃ¼rk, G.	2013	Clinical experience in the surgical treatment of primary hyperparathyroidism: 94 cases	Duzce Medical Journal	Not English

22	Babinska, Dominika; Barczynski, Marcin; Stefaniak, Tomasz; Oseka, Tomasz; Babinska, Anna; Babinski, Dariusz; Sworczak, Krzysztof; Lachinski, Andrzej J.; Nowak, Wojciech; Sledzinski, Zbigniew	2012	Evaluation of selected cognitive functions before and after surgery for primary hyperparathyroidism	Langenbeck's archives of surgery	No measurement properties
23	Bandeira, Francisco; Griz, Luiz; Caldas, Gustavo; Bandeira, Cristina; Freese, Eduardo	2006	From mild to severe primary hyperparathyroidism: The Brazilian experience	Arquivos brasileiros de endocrinologia e metabologia	PROM not used
24	Bandeira, Leonardo; Bilezikian, John	2016	Primary Hyperparathyroidism	F1000Research	Review/Guideline
25	Bannani, S.; Christou, N.; Guerin, C.; Hamy, A.; Sebag, F.; Mathonnet, M.; Guillot, P.; Caillard, C.; Blanchard, C.; Mirallie, E.	2018	Effect of parathyroidectomy on quality of life and non-specific symptoms in normocalcaemic primary hyperparathyroidism	The British journal of surgery	No measurement properties
26	Bargren, Anna E.; Repplinger, Daniel; Chen, Herbert; Sippel, Rebecca S.	2011	Can biochemical abnormalities predict symptomatology in patients with primary hyperparathyroidism?	Journal of the American College of Surgeons	PROM not used
27	Barker, Holly; Caldwell, Lauren; Lovato, James; Woods, Kristy F.; Perrier, Nancy D.	2004	Is there a racial difference in presentation of primary hyperparathyroidism?	The American surgeon	PROM not used
28	Barkun, J.; Duh, Q. Y.; Wiseman, S.; McKenzie, M.; Evidence Surgery, Grp	2006	Canadian association of general surgeons and American College of Surgeons evidence based reviews in surgery. 16 - Randomized trial of parathyroidectomy in mild asymptomatic primary hyperparathyroidism	CANADIAN JOURNAL OF SURGERY	Review/Guideline
29	Baugh, K; Liu, J; Yip, L; McCoy, K; Carty, S; Ramonell, K	2023	Sex differences in patients with primary hyperparathyroidism	Surgery	No measurement properties
30	Bednarek-Tupikowska, G.; Tupikowski, K.	2008	The role of primary care physician in the diagnosis of primary hyperparathyroidism	Family Medicine and Primary Care Review	Review/Guideline
31	Bell, Caitlin F.; Warrick, Mathew M.; Gallagher, Kathleen C.; Baregamian, Naira	2018	Neurocognitive performance profile postparathyroidectomy: a pilot study of computerized assessment	Surgery	PROM not used

32	Benge, Jared F.; Perrier, Nancy D.; Massman, Paul J.; Meyers, Christina A.; Kayl, Anne E.; Wefel, Jeffrey S.	2009	Cognitive and affective sequelae of primary hyperparathyroidism and early response to parathyroidectomy	Journal of the International Neuropsychological Society : JINS	No measurement properties
33	Bhadada, Sanjay Kumar; Arya, Ashutosh Kumar; Mukhopadhyay, Satinath; Khadgawat, Rajesh; Sukumar, Suja; Lodha, Sailesh; Singh, Deependra N.; Sathya, Anjali; Singh, Priyanka; Bhansali, Anil	2018	Primary hyperparathyroidism: insights from the Indian PHPT registry	Journal of bone and mineral metabolism	PROM not used
34	Bilezikian, J. P.; Brandi, M. L.; Rubin, M.; Silverberg, S. J.	2005	Primary hyperparathyroidism: new concepts in clinical, densitometric and biochemical features	Journal of internal medicine	Review/Guideline
35	Bilezikian, J. P.; Khan, A. A.; Silverberg, S. J.; Fuleihan, G. E. H.; Marcocci, C.; Minisola, S.; Perrier, N.; Sitges-Serra, A.; Thakker, R. V.; Guyatt, G.; Mannstadt, M.; Potts, J. T.; Clarke, B. L.; Brandi, M. L.	2022	Evaluation and Management of Primary Hyperparathyroidism: Summary Statement and Guidelines from the Fifth International Workshop	Journal of Bone and Mineral Research	Review/Guideline
36	Bilezikian, John P.	2018	Primary Hyperparathyroidism	The Journal of clinical endocrinology and metabolism	Review/Guideline
37	Bilezikian, John P.; Bandeira, Leonardo; Khan, Aliya; Cusano, Natalie E.	2018	Hyperparathyroidism	Lancet (London, England)	Review/Guideline
38	Birkenhager, J. C.; Bouillon, R.	1996	Asymptomatic primary hyperparathyroidism	Postgraduate medical journal	Review/Guideline
39	Blanchard, C.; Mirallie, E.; Mathonnet, M.	2010	Sporadic primary hyperparathyroidism	Journal of visceral surgery	Review/Guideline

40	Blanchard, Claire; Mathonnet, Muriel; Sebag, Frederic; Caillard, Cecile; Hamy, Antoine; Volteau, Christelle; Heymann, Marie-Francoise; Wyart, Vincent; Drui, Delphine; Roy, Malanie; Cariou, Bertrand; Archambeaud, Francoise; Rodien, Patrice; Henry, Jean-Francois; Zarnegar, Rasa; Hardouin, Jean-Benoit; Mirallie, Eric	2013	Surgery for 'asymptomatic' mild primary hyperparathyroidism improves some clinical symptoms postoperatively	European journal of endocrinology	PROM not used
41	Blanchard, Claire; Mathonnet, Muriel; Sebag, Frederic; Caillard, Cecile; Kubis, Caroline; Drui, Delphine; van Nuvel, Elise; Ansquer, Catherine; Henry, Jean-Francois; Masson, Damien; Kraeber-Bodere, Francoise; Hardouin, Jean-Benoit; Zarnegar, Rasa; Hamy, Antoine; Mirallie, Eric	2014	Quality of life is modestly improved in older patients with mild primary hyperparathyroidism postoperatively: results of a prospective multicenter study	Annals of surgical oncology	No measurement properties
42	Bollerslev, J.; Ã˜gard, C. G.; Schwarz, P.; Vestergaard, H.; Vestergaard, P.	2005	Primary hyperparathyroidism: Occurrence, symptoms, complications and treatment	Ugeskrift for Laeger	Review/Guideline
43	Bollerslev, Jens; Jansson, Svante; Mollerup, Charlotte L.; Nordenstrom, Jorgen; Lundgren, Eva; Torring, Ove; Varhaug, Jan-Erik; Baranowski, Marek; Aanderud, Sylvi; Franco, Celina; Freyschuss, Bo; Isaksen, Gunhild A.; Ueland, Thor; Rosen, Thord	2007	Medical observation, compared with parathyroidectomy, for asymptomatic primary hyperparathyroidism: a prospective, randomized trial	The Journal of clinical endocrinology and metabolism	No measurement properties

44	Bollerslev, Jens; Marcocci, Claudio; Sosa, Manuel; Nordenstrom, Jorgen; Bouillon, Roger; Mosekilde, Leif	2011	Current evidence for recommendation of surgery, medical treatment and vitamin D repletion in mild primary hyperparathyroidism	European journal of endocrinology	Review/Guideline
45	Boone, Deva; Politz, Douglas; Lopez, Jose; Mitchell, Jamie; Parrack, Kevin; Norman, James	2017	Concentration of serum calcium is not correlated with symptoms or severity of primary hyperparathyroidism: An examination of 20,081 consecutive adults	Surgery	PROM not used
46	Brescia, Marilia D'Elboux Guimaraes; Rodrigues, Karine Candido; d'Alessandro, Andre Fernandes; Alves Filho, Wellington; van der Plas, Willemijn Y.; Kruijff, Schelto; Arap, Sergio Samir; Toledo, Sergio Pereira de Almeida; Montenegro, Fabio Luiz de Menezes; Lourenco, Delmar Muniz, Jr.	2022	Impact of parathyroidectomy on quality of life in multiple endocrine neoplasia type 1	Endocrine connections	No measurement properties
47	Brito, Kenneth; Edirimanne, Senarath; Eslick, Guy D.	2015	The extent of improvement of health-related quality of life as assessed by the SF36 and Paseika scales after parathyroidectomy in patients with primary hyperparathyroidism--a systematic review and meta-analysis	International journal of surgery (London, England)	Review/Guideline
48	Brothers, T. E.; Thompson, N. W.	1987	Surgical treatment of primary hyperparathyroidism in elderly patients	Acta chirurgica Scandinavica	PROM not used
49	Brown, G. G.; Preisman, R. C.; Kleerekoper, M.	1987	Neurobehavioral symptoms in mild primary hyperparathyroidism: related to hypercalcemia but not improved by parathyroidectomy	Henry Ford Hospital medical journal	PROM not used
50	Burney, Richard E.	2011	Cognitive improvement after parathyroidectomy	Annals of surgery	Not primary research study
51	CÃZares Robles, Tania K.; FernÃNdez-Posada De La Mora, RocÃO; GÃ“Mez-MartÃNez, Manuel A.; RodrÃGuez-GÃ“Mez, Adriana; Rojas-JimÃ‰Nez, Ernesto A.	2021	Biochemistry in Symptomatic and Asymptomatic Primary Hyperparathyroidism: A Cross-Sectional Study	Revista Mexicana de EndocrinologÃ-a, Metabolismo y NutriciÃ³n	PROM not used

52	Caillard, Cecile; Sebag, Frederic; Mathonnet, Muriel; Gibelin, Helene; Brunaud, Laurent; Loudot, Coralie; Kraimps, Jean-Louis; Hamy, Antoine; Bresler, Laurent; Charbonnel, Bernard; Leborgne, Joel; Henry, Jean-Francois; Nguyen, Jean-Michel; Mirallie, Eric	2007	Prospective evaluation of quality of life (SF-36v2) and nonspecific symptoms before and after cure of primary hyperparathyroidism (1-year follow-up)	Surgery	No measurement properties
53	Campbell, M. J.	2017	The definitive management of primary hyperparathyroidism who needs an operation?	JAMA - Journal of the American Medical Association	Review/Guideline
54	Carnevale, V.; Romagnoli, E.; Pipino, M.; Scillitani, A.; D'Erasmo, E.; Minisola, S.; Mazzuoli, G.	2005	[Primary hyperparathyroidism]	Iperparatiroidismo primitivo.	Review/Guideline
55	Caron, Nadine R.; Pasieka, Janice L.	2009	What symptom improvement can be expected after operation for primary hyperparathyroidism?	World journal of surgery	Review/Guideline
56	Casella, Claudio; Pata, Giacomo; Di Betta, Ernesto; Nascimbeni, Riccardo	2008	[Neurological and psychiatric disorders in primary hyperparathyroidism: the role of parathyroidectomy]	Manifestazioni neuropsichiche in corso di iperparatiroidiso primitivo: il ruolo della paratiroidectomia.	Not English
57	Caton, Nadine Louise; Choudhury, Natasha	2019	Quality of life improvement following parathyroid surgery: A preliminary 3â€year review of 56 patients from a single surgical centre	Clin Otolaryngol	No measurement properties
58	Chan, A. K.; Duh, Q. Y.; Katz, M. H.; Siperstein, A. E.; Clark, O. H.	1995	Clinical manifestations of primary hyperparathyroidism before and after parathyroidectomy. A case-control study	Annals of surgery	PROM not used
59	Chandran, Manju; Yeh, Lydia Tan Li; de Jong, Mechteld C.; Bilezikian, John P.; Parameswaran, Rajeev	2022	Cognitive deficits in primary hyperparathyroidism - what we know and what we do not know: A narrative review	Reviews in endocrine & metabolic disorders	Review/Guideline
60	Chen, Herbert	2020	Parathyroid Disease: Often Forgotten and Undertreated	American Surgeon	Review/Guideline

61	Chen, Herbert; Parkerson, Sara; Udelsman, Robert	1998	Parathyroidectomy in the Elderly: Do the Benefits Outweigh the Risks?	World J. Surg.	PROM not used
62	Cheng, Shih-Ping; Lee, Jie-Jen; Liu, Tsang-Pai; Yang, Po-Sheng; Liu, Sung-Chen; Hsu, Yi-Chiung; Liu, Chien-Liang	2015	Quality of Life After Surgery or Surveillance for Asymptomatic Primary Hyperparathyroidism: A Meta-Analysis of Randomized Controlled Trials	Medicine	Review/Guideline
63	Chiang, Cherie Y.; Andrewes, David G.; Anderson, Dianne; Devere, Michael; Schweitzer, Isaac; Zajac, Jeffrey D.	2005	A controlled, prospective study of neuropsychological outcomes post parathyroidectomy in primary hyperparathyroid patients	Clinical endocrinology	No measurement properties
64	Chiba, Yuko; Satoh, Katsuhiko; Ueda, Satoshi; Kanazawa, Nobuo; Tamura, Yoshiaki; Horiuchi, Toshiyuki	2007	Marked improvement of psychiatric symptoms after parathyroidectomy in elderly primary hyperparathyroidism	Endocrine journal	No measurement properties
65	Chigot, J. P.; Menegaux, F.; Achrafi, H.	1995	Should primary hyperparathyroidism be treated surgically in elderly patients older than 75 years?	Surgery	PROM not used
66	Chigot, J. P.; Menegaux, F.; Dahman, M.; Schmitt, G.	1998	Primary hyperparathyroidism in elderly patients. A retrospective study of 115 operated patients	Revue Francaise d'Endocrinologie Clinique - Nutrition et Metabolisme	PROM not used
67	Christensen, Julie Wulf; Thogersen, Karin Folmer; Jensen, Lars Thorbjorn; Krakauer, Martin; Kristensen, Bent; Bennedbaek, Finn Noe; Zerahn, Bo	2022	Changes in quality of life 6 months after parathyroidectomy for primary hyperparathyroidism	Endocrine connections	No measurement properties
68	Cipriani, Cristiana; Cianferotti, Luisella	2022	Quality of Life in Primary Hyperparathyroidism	Endocrinology and metabolism clinics of North America	Review/Guideline
69	Cipriani, Cristiana; Romagnoli, Elisabetta; Cilli, Mirella; Piemonte, Sara; Pepe, Jessica; Minisola, Salvatore	2014	Quality of life in patients with primary hyperparathyroidism	Expert review of pharmacoeconomics & outcomes research	Review/Guideline

70	Clark, O. H.; Wilkes, W.; Siperstein, A. E.; Duh, Q. Y.	1991	Diagnosis and management of asymptomatic hyperparathyroidism: safety, efficacy, and deficiencies in our knowledge	Journal of bone and mineral research : the official journal of the American Society for Bone and Mineral Research	PROM not used
71	Coker, Laura H.; Rorie, Kashemi; Cantley, Larry; Kirkland, Kimberly; Stump, David; Burbank, Nicole; Tembreull, Terry; Williamson, Jeff; Perrier, Nancy	2005	Primary hyperparathyroidism, cognition, and health-related quality of life	Annals of surgery	Review/Guideline
72	Conroy, Simon; Moulias, Sophie; Wassif, Wassif S.	2003	Primary hyperparathyroidism in the older person	Age and ageing	Review/Guideline
73	Coston, S. D.; Pelton, J. J.	1999	Success of cervical exploration for patients with asymptomatic primary hyperparathyroidism	American journal of surgery	PROM not used
74	Dawood, Nardeen B.; Yan, Kimberly L.; Shieh, Albert; Livhits, Masha J.; Yeh, Michael W.; Leung, Angela M.	2020	Normocalcaemic primary hyperparathyroidism: An update on diagnostic and management challenges	Clinical endocrinology	Review/Guideline
75	Delbridge, L. W.; Marshman, D.; Reeve, T. S.; Crummer, P.; Posen, S.	1988	Neuromuscular symptoms in elderly patients with hyperparathyroidism: improvement with parathyroid surgery	The Medical journal of Australia	PROM not used
76	Deutch, Soren; Jensen, Martin; Christiansen, Peer; Hessov; D.M.	2000	Muscular Performance and Fatigue in Primary Hyperparathyroidism	World journal of surgery	PROM not used
77	Djafarrian, Reza; Laurent, Margaux; Demarchi, Marco; Bianchetto Wolf, Nicola; Luzuy-Guarnero, Valentine; Zingg, Tobias; Matter, Maurice; Triponez, Frederic	2023	[Surgical management of primary hyperparathyroidism]	Prise en charge chirurgicale de l'hyperparathyroidie primaire.	Review/Guideline

78	Dotzenrath, Cornelia M. E.; Kaetsch, Ana Karena; Pfingsten, Henrich; Cupisti, Kenko; Weyerbrock, Norbert; Vossough, Alexander; Verde, Pablo E.; Ohmann, Christian	2006	Neuropsychiatric and cognitive changes after surgery for primary hyperparathyroidism	World journal of surgery	No measurement properties
79	Dulfer, Roderick; Geilvoet, Wanda; Morks, Annelien; van Lieshout, Esther M. M.; Smit, Casper P. C.; Nieveen van Dijkum, Els J. M.; In't Hof, Klaas; van Dam, Frits; van Eijck, Casper H. J.; de Graaf, Peter W.; van Ginhoven, Tessa M.	2016	Impact of parathyroidectomy for primary hyperparathyroidism on quality of life: A case-control study using Short Form Health Survey 36	Head & neck	No measurement properties
80	Edwards, M. E.; Rotramel, A.; Beyer, T.; Gaffud, M. J.; Djuricin, G.; Loviscek, K.; Solorzano, C. C.; Prinz, R. A.	2006	Improvement in the health-related quality-of-life symptoms of hyperparathyroidism is durable on long-term follow-up	Surgery	No measurement properties
81	Egan, Kelly R.; Adler, Joel T.; Olson, Jordan E.; Chen, Herbert	2007	Parathyroidectomy for primary hyperparathyroidism in octogenarians and nonagenarians: a risk-benefit analysis	The Journal of surgical research	No measurement properties
82	Eigelberger, Monica S.; Cheah, W. Keat; Ituarte, Philip H. G.; Streja, Leanne; Duh, Quan-Yang; Clark, Orlo H.	2004	The NIH criteria for parathyroidectomy in asymptomatic primary hyperparathyroidism: are they too limited?	Annals of surgery	PROM not used
83	Ejlsmark-Svensson, Henriette; Sikjaer, Tanja; Webb, Susan M.; Rejnmark, Lars; Rolighed, Lars	2019	Health-related quality of life improves 1 year after parathyroidectomy in primary hyperparathyroidism: A prospective cohort study	Clinical endocrinology	No measurement properties
84	Elaraj, Dina M.; Clark, Orlo H.	2008	Current status and treatment of primary hyperparathyroidism	The Permanente journal	Review/Guideline

85	El-Hajj Fuleihan, Ghada; Chakhtoura, Marlene; Cipriani, Cristiana; Eastell, Richard; Karonova, Tatiana; Liu, Jian-Min; Minisola, Salvatore; Mithal, Ambrish; Moreira, Carolina A.; Peacock, Munro; Schini, Marian; Silva, Barbara; Walker, Marcella; El Zein, Ola; Marcocci, Claudio	2022	Classical and Nonclassical Manifestations of Primary Hyperparathyroidism	Journal of bone and mineral research : the official journal of the American Society for Bone and Mineral Research	Review/Guideline
86	Espiritu, Rachel P.; Kearns, Ann E.; Vickers, Kristin S.; Grant, Clive; Ryu, Euijung; Wermers, Robert A.	2011	Depression in primary hyperparathyroidism: prevalence and benefit of surgery	The Journal of clinical endocrinology and metabolism	No measurement properties
87	Euctr, I. T.	2014	IMPACT OF VITAMIN D SUPPLEMENTATION ON SKELETAL AND NON SKELETAL MANIFESTATIONS IN PATIENTS WITH PRIMARY HYPERPARATHYROIDISM SUBMITTED TO PARATHYROIDECTOMY OR FOLLOWED WITHOUT SURGERY	https://trialsearch.who.int/Trial2.aspx?TrialID=EUCTR2013-005027-16-IT	Trial protocol
88	Euctr, I. T.	2010	TREATMENT OF HYPERPARATHYROIDISM IN PATIENTS WITH MULTIPLE ENDOCRINE NEOPLASIA TYPE 1 (MEN1) WITH THE CALCIMIMETIC AGENT CINACALCET - ND	https://trialsearch.who.int/Trial2.aspx?TrialID=EUCTR2008-005055-23-IT	Trial protocol
89	Euctr, S. E.	2012	Use of calcimimetics in primary hyperparathyroidism to predict the outcome of parathyroid surgery	https://trialsearch.who.int/Trial2.aspx?TrialID=EUCTR2012-005374-57-SE	Trial protocol
90	Febrero, Beatriz; Ruiz-Manzanera, Juan Jose; Ros-Madrid, Inmaculada; Hernandez-Martinez, Antonio-Miguel; Rodriguez, Jose M.	2023	The Influence of Hyperparathyroidism Patient Profile on Quality of Life After Parathyroidectomy	World journal of surgery	No measurement properties
91	Fenech, M. E.; Turner, J. J. O.	2013	Hypercalcaemia and primary hyperparathyroidism	Medicine (United Kingdom)	Review/Guideline

92	Flint, Richard S.; Harman, Christopher Richard; Carter, Jim; Snyman, Gerrie	2002	Primary hyperparathyroidism: referral patterns and outcomes of surgery	ANZ journal of surgery	PROM not used
93	Frey, Samuel; Perrot, Bastien; Caillard, Cecile; Le Bras, Maelle; Gerard, Maxime; Blanchard, Claire; Cariou, Bertrand; Wargny, Matthieu; Mirallie, Eric	2023	Parathyroidectomy for primary hyperparathyroidism: effect on quality of life after 3 years - a prospective cohort study	International journal of surgery (London, England)	No measurement properties
94	Gasser, Rudolf Wolfgang	2013	Clinical aspects of primary hyperparathyroidism: clinical manifestations, diagnosis, and therapy	Wiener medizinische Wochenschrift (1946)	Review/Guideline
95	Gladkova, I. N.; Rusakov, V. F.; Chernikov, R. A.; Karelina, Yu V.; Nikitina, T. P.; Efremov, S. M.; Ionova, T. I.	2021	[Validation and testing of the Russian version of PHPQoL questionnaire for quality of life assessment in patients with primary hyperparathyroidism (PHPT)]	Problemy endokrinologii	Not English
96	Gladkova, I.; Chernikov, R.; Efremov, S.; Ionova, T.; Nikitina, T.; Rusakov, V.; Shablovskaya, N.	2020	Quality of life in patients with primary hyperparathyroidism after parathyroidectomy	QUALITY OF LIFE RESEARCH	Not primary research study
97	Gopinath, Preethi; Sadler, Gregory P.; Mihai, Radu	2010	Persistent symptomatic improvement in the majority of patients undergoing parathyroidectomy for primary hyperparathyroidism	Langenbeck's archives of surgery	No measurement properties
98	Goyal, A.; Chumber, S.; Tandon, N.; Lal, R.; Srivastava, A.; Gupta, S.	2001	Neuropsychiatric manifestations in patients of primary hyperparathyroidism and outcome following surgery	Indian journal of medical sciences	No measurement properties
99	Grant, Paul; Velusamy, Anand	2014	What is the best way of assessing neurocognitive dysfunction in patients with primary hyperparathyroidism?	The Journal of clinical endocrinology and metabolism	Review/Guideline
100	Greutelaers, Benedikt; Kullen, Katrin; Kollias, James; Bochner, Melissa; Roberts, Anthony; Wittert, Garry; Pasieka, Janice; Malycha, Peter	2004	Pasieka Illness Questionnaire: its value in primary hyperparathyroidism	ANZ journal of surgery	No measurement properties
101	Habib, Zeina; Camacho, Pauline	2010	Primary hyperparathyroidism: an update	Current opinion in endocrinology, diabetes, and obesity	Review/Guideline

102	Hargitai, L.; Clerici, T.; Musholt, T. J.; Riss, P.; Eurocrine council	2023	Surgery for primary hyperparathyroidism in Germany, Switzerland, and Austria: an analysis of data from the EUROCRINE registry	Langenbeck's archives of surgery	PROM not used
103	Harrington, Candace C.; Hayden, Dedra M.	2023	Maximizing Opportunities: Primary Hyperparathyroidism in the Older Adult	Journal for Nurse Practitioners	Review/Guideline
104	Hasse, Christian; Sitter, Helmut; Brune, Melanie; Wollenteit, Ina; Nies, Christoph; Rothmund, Matthias	2002	Quality of life and patient satisfaction after reoperation for primary hyperparathyroidism: analysis of long-term results	World journal of surgery	No measurement properties
105	Heath, D. A.	1989	Primary hyperparathyroidism. Clinical presentation and factors influencing clinical management	Endocrinology and metabolism clinics of North America	Review/Guideline
106	Heath, V.	2009	Parathyroid Function: Cognitive dysfunction in women with mild primary hyperparathyroidism	Nature Reviews Endocrinology	Not primary research study
107	Hedback, Gunilla; Oden, Anders	2004	Persistent disease after surgery for primary hyperparathyroidism: the long-term outcome	European journal of endocrinology	PROM not used
108	Hermsen, A.; Eienbroker, A.; Haag, A.; Mylius, V.; Hamer, H. M.; Menzler, K.; Karakas, E.; Rosenow, F.	2014	Perioperative changes in cortical excitability, mood, and quality of life in patients with primary hyperparathyroidism: a pilot study using transcranial magnetic stimulation	European journal of endocrinology	No measurement properties
109	Horiuchi, Kiyomi; Yoshida, Yusaku; Okamoto, Takahiro	2020	Effects of surgery on the patient-reported outcomes of primary hyperparathyroidism patients with mild hypercalcemia without classic symptoms: a systematic review of the literature	Surgery today	Review/Guideline
110	Ionova, T. I.; Buzanakov, D. M.; Chernikov, R. A.; Efremov, S. M.; Gladkova, I. N.; Nikitina, T. P.; Sleptsov, I. V.; Zolotoukho, A. V.; Bubnov, K. A.; Skvortsov, V. V.; Vinogradova, A. A.; Rusakov, V. F.	2023	Quality of life in patients with primary hyperparathyroidism before and after parathyroidectomy: long term single center experience	BMC endocrine disorders	No measurement properties
111	Islam, Ana Kashfia	2021	Advances in the diagnosis and the management of primary hyperparathyroidism	Therapeutic advances in chronic disease	Review/Guideline

112	Jannasch, O.; Voigt, C.; Klose, S.; Meyer, F.; Mroczkowski, P.	2016	[Unicentre Results in Surgery of Primary Hyperparathyroidism with Postoperative Long-Term Follow-Up and Value of Intraoperative Quick Parathormone Test]	Unizentrische Ergebnisse der Chirurgie des primaren Hyperparathyreoidismus mit postoperativer Langzeitbeobachtung sowie Rolle des intraoperativen Quick-Parathormon-Tests.	Not English
113	Jansson, Svante; Morgan, Eric	2004	Biochemical effects from treatment with bisphosphonate and surgery in patients with primary hyperparathyroidism	World journal of surgery	PROM not used
114	Jarhult, Johannes	2008	Prospective evaluation of symptom responses to successful operation for mild-moderate primary hyperparathyroidism	Scandinavian journal of surgery : SJS : official organ for the Finnish Surgical Society and the Scandinavian Surgical Society	PROM not used
115	Joborn, C.; Hetta, J.; Lind, L.; Rastad, J.; Akerstrom, G.; Ljunghall, S.	1989	Self-rated psychiatric symptoms in patients operated on because of primary hyperparathyroidism and in patients with long-standing mild hypercalcemia	Surgery	No measurement properties
116	Joborn, C.; Hetta, J.; Palmer, M.; Akerstrom, G.; Ljunghall, S.	1986	Psychiatric symptomatology in patients with primary hyperparathyroidism	Upsala journal of medical sciences	PROM not used
117	Joborn, C.; Hetta, J.; Rastad, J.; Agren, H.; Akerstrom, G.; Ljunghall, S.	1988	Psychiatric symptoms and cerebrospinal fluid monoamine metabolites in primary hyperparathyroidism	Biological psychiatry	No measurement properties
118	Jovanovic, Milan; Zivaljevic, Vladan; Sipetic Grujicic, Sandra; Tausanovic, Katarina; Slijepcevic, Nikola; Rovcanin, Branislav; Jovanovic, Ksenija; Odalovic, Bozidar; Buzejic, Matija; Bukumiric, Zoran; Paunovic, Ivan	2023	Effects of successful parathyroidectomy on neuropsychological and cognitive status in patients with asymptomatic primary hyperparathyroidism	Endocrine	No measurement properties

119	Kahal, Hassan; Aye, Mo; Rigby, Alan S.; Sathyapalan, Thozhukat; England, R. J. A.; Atkin, Stephen L.	2012	The effect of parathyroidectomy on neuropsychological symptoms and biochemical parameters in patients with asymptomatic primary hyperparathyroidism	Clinical endocrinology	No measurement properties
120	Karwacki, J. H.; Skalski, A.; Nawrot, I.	2007	Analysis of prior medical histories of patients operated on for primary hyperparathyroidism	Advances in Clinical and Experimental Medicine	PROM not used
121	Kearns, Ann E.; Espiritu, Rachel P.; Vickers Douglass, Kristin; Thapa, Prabin; Wermers, Robert A.	2019	Clinical characteristics and depression score response after parathyroidectomy in primary hyperparathyroidism	Clinical endocrinology	No measurement properties
122	Kebebew, Electron; Duh, Quan-Yang; Clark, Orlo H.	2003	Parathyroidectomy for primary hyperparathyroidism in octogenarians and nonagenarians: a plea for early surgical referral	Archives of surgery (Chicago, Ill. : 1960)	PROM not used
123	Khan, A. A.; Bilezikian, J. P.; Bone, H. G.; Gurevich, A.; Lakatos, P.; Misiorowski, W.; Rozhinskaya, L.; Trotman, M. L.; Toth, M.	2014	Cinacalcet normalizes serum calcium in a randomized, placebocontrolled clinical study in patients with primary hyperparathyroidism unable to undergo parathyroidectomy	Endocrine reviews	No measurement properties
124	Khan, A. A.; Hanley, D. A.; Rizzoli, R.; Bollerslev, J.; Young, J. E. M.; Rejnmark, L.; Thakker, R.; D'Amour, P.; Paul, T.; Van Uum, S.; Shrayyef, M. Zakaria; Goltzman, D.; Kaiser, S.; Cusano, N. E.; Bouillon, R.; Mosekilde, L.; Kung, A. W.; Rao, S. D.; Bhadada, S. K.; Clarke, B. L.; Liu, J.; Duh, Q.; Lewiecki, E. Michael; Bandeira, F.; Eastell, R.; Marcocci, C.; Silverberg, S. J.; Udelsman, R.; Davison, K. Shawn; Potts, J. T., Jr.; Brandi, M. L.; Bilezikian, J. P.	2017	Primary hyperparathyroidism: review and recommendations on evaluation, diagnosis, and management. A Canadian and international consensus	Osteoporosis international : a journal established as result of cooperation between the European Foundation for Osteoporosis and the National Osteoporosis Foundation of the USA	Review/Guideline

125	Khan, Aliya A.	2013	Medical management of primary hyperparathyroidism	Journal of clinical densitometry : the official journal of the International Society for Clinical Densitometry	Review/Guideline
126	Kobayashi, T.; Sugimoto, T.; Chihara, K.	1997	Clinical and biochemical presentation of primary hyperparathyroidism in Kansai district of Japan	Endocrine journal	PROM not used
127	Kolyvanos Naumann, U.; Kaser, L.; Vetter, W.	2004	[Primary hyperparathyroidism. Main symptoms: fatigue, weakness, lack of appetite, constipation, mental disorders, bone pain]	Primarer Hyperparathyreoidismus. Leitsymptome: Mudigkeit, Schwache, Inappetenz, Obstipation, psychische Storungen, Knochenschmerzen.	Not English
128	Koman, A.; Branstrom, R.; Pernow, Y.; Branstrom, R.; Nilsson, I. L.	2021	Prediction of cognitive response to surgery in elderly patients with primary hyperparathyroidism	BJS open	No measurement properties
129	Koman, A.; Branstrom, R.; Pernow, Y.; Branstrom, R.; Nilsson, I. L.	2020	COGNITIVE DEFICIENCY IN ELDERLY WITH PRIMARY HYPERPARATHYROIDISM; SHOULD ASSESSMENT BE MODIFIED TO BETTER PREDICT THE EFFECTS AFTER PARATHYROIDECTOMY?	British Journal of Surgery	No measurement properties
130	Koman, A.; Ohlsson, S.; Branstrom, R.; Pernow, Y.; Branstrom, R.; Nilsson, I. L.	2019	Short-term medical treatment of hypercalcaemia in primary hyperparathyroidism predicts symptomatic response after parathyroidectomy	The British journal of surgery	No measurement properties
131	Kristoffersson, A.; Dahlgren, K.; Granstrand, B.; Jarhult, J.	1987	Primary hyperparathyroidism in Northern Sweden	Surgery, gynecology & obstetrics	PROM not used
132	Kunert, Å; SoÅ,tysik, M.; BuÅ,a, G.; Gawrychowski, J.; Pudlo, R.	2016	Mental disorders in patients with primary hyperparathyroidism	Psychiatria	Review/Guideline
133	La, Justin; Wang, Tracy S.; Hammad, Abdulrahman Y.; Burgardt, Laura; Doffek, Kara; Carr, Azadeh A.; Shaker, Joseph L.; Carroll, Ty B.; Evans, Douglas B.; Yen, Tina W. F.	2017	Parathyroidectomy for primary hyperparathyroidism improves sleep quality: A prospective study	Surgery	No measurement properties

134	Leong, K. J.; Sam, R. C.; Garnham, A. W.	2010	Health-related quality of life improvement following surgical treatment of primary hyperparathyroidism in a United Kingdom population	The surgeon : journal of the Royal Colleges of Surgeons of Edinburgh and Ireland	No measurement properties
135	Linder, J.; Brimar, K.; Granberg, P. O.; Wetterberg, L.; Werner, S.	1988	Characteristic changes in psychiatric symptoms, cortisol and melatonin but not prolactin in primary hyperparathyroidism	Acta psychiatrica Scandinavica	No measurement properties
136	Liu, Huijiang; Luo, Kai; Liao, Shijie; Tang, Haijun; Mo, Jianming; Xie, Tianyu; Li, Chong; Li, Boxiang; Liu, Yun; Zhan, Xinli	2022	Diagnosis and treatment of primary hyperparathyroidism with pathological fracture of the limbs: A retrospective observational study	Medicine	PROM not used
137	Liu, Jessica Y.; Peine, Brandon S.; Mlaver, Eli; Patel, Snehal G.; Weber, Collin J.; Saunders, Neil D.; Pofahl, Walter E.; Sharma, Jyotirmay	2021	Neuropsychologic changes in primary hyperparathyroidism after parathyroidectomy from a dual-institution prospective study	Surgery	No measurement properties
138	Liu, Jessica Y.; Saunders, Neil D.; Chen, Aaron; Weber, Collin J.; Sharma, Jyotirmay	2016	Neuropsychological Changes in Primary Hyperparathyroidism after Parathyroidectomy	The American surgeon	No measurement properties
139	Liu, M.; Sum, M.; Cong, E.; Colon, I.; Bucovsky, M.; Williams, J.; Kepley, A.; Kuo, J.; Lee, J. A.; Lazar, R. M.; Marshall, R.; Silverberg, S.; Walker, M. D.	2020	Cognition and cerebrovascular function in primary hyperparathyroidism before and after parathyroidectomy	Journal of endocrinological investigation	No measurement properties
140	Liu, Y. A.; Guo, S. Y.; Wu, J. S.; Wang, R. G.; Liu, J. B.; Liu, Y.; Bin, L.; Liu, N.; Jiang, L.; Zhang, X. L.	2021	Changes in clinical patterns of Chinese patients with primary hyperparathyroidism in the past 12 years: a single-center experience	Endocrine connections	PROM not used
141	Livschitz, Jennifer; Yen, Tina W. F.; Evans, Douglas B.; Wang, Tracy S.; Dream, Sophie	2022	Long-term Quality of Life After Parathyroidectomy for Primary Hyperparathyroidism: A Systematic Review	JAMA surgery	Review/Guideline
142	Ljunghall, S.; Jakobsson, S.; Joborn, C.; Palmer, M.; Rastad, J.; Akerstrom, G.	1991	Longitudinal studies of mild primary hyperparathyroidism	Journal of bone and mineral research : the official journal of the American Society for Bone and Mineral Research	No measurement properties

143	Lumachi, Franco; Basso, Stefano M. M.	2014	Pathophysiology and treatment of nonfamilial hyperparathyroidism	Recent patents on CNS drug discovery	Review/Guideline
144	Lundgren, E.; Ljunghall, S.; AkerstrÃ¶m, G.; Hetta, J.; Mallmin, H.; Rastad, J.	1998	Case-control study on symptoms and signs of "asymptomatic" primary hyperparathyroidism	Surgery	No measurement properties
145	Lundgren, E.; Werner, S.; Farnebo, L. O.; Bollerslev, J.; Akerstrom, G.	1999	[A Scandinavian multicenter study will investigate the question: surgery or not in mild primary hyperparathyroidism?]	Skandinavisk multicenterstudie skall utreda fragan: kirurgi eller ej vid lindrig primar hyperparatyreoidism?	Not English
146	Macfarlane, David P.; Yu, Ning; Leese, Graham P.	2013	Subclinical and asymptomatic parathyroid disease: implications of emerging data	The lancet. Diabetes & endocrinology	Review/Guideline
147	Machado, Nikita N.; Wilhelm, Scott M.	2019	Diagnosis and Evaluation of Primary Hyperparathyroidism	The Surgical clinics of North America	Review/Guideline
148	Mack, L. A.; Pasieka, J. L.	2007	The spectrum of clinical benefits following parathyroidectomy for primary hyperparathyroidism	Clinical Reviews in Bone and Mineral Metabolism	Review/Guideline
149	Mack, Lloyd A.; Pasieka, Janice L.	2004	Asymptomatic primary hyperparathyroidism: a surgical perspective	The Surgical clinics of North America	Review/Guideline
150	Marcocci, Claudio; Chanson, Philippe; Shoback, Dolores; Bilezikian, John; Fernandez-Cruz, Laureano; Orgiazzi, Jacques; Henzen, Christoph; Cheng, Sunfa; Sterling, Lulu Ren; Lu, John; Peacock, Munro	2009	Cinacalcet Reduces Serum Calcium Concentrations in Patients with Intractable Primary Hyperparathyroidism	The Journal of clinical endocrinology and metabolism	PROM not used
151	McAllion, S. J.; Paterson, C. R.	1989	Psychiatric morbidity in primary hyperparathyroidism	Postgraduate medical journal	PROM not used
152	McDow, Alexandria D.; Sippel, Rebecca S.	2018	Should Symptoms Be Considered an Indication for Parathyroidectomy in Primary Hyperparathyroidism?	Clinical medicine insights. Endocrinology and diabetes	Review/Guideline
153	Mihai, Radu; Wass, John A. H.; Sadler, Gregory P.	2008	Asymptomatic hyperparathyroidism--need for multicentre studies	Clinical endocrinology	Review/Guideline

154	Milat, Frances; Ramchand, Sabashini K.; Herath, Madhuni; Gundara, Justin; Harper, Simon; Farrell, Stephen; Girgis, Christian M.; Clifton-Bligh, Roderick; Schneider, Hans G.; De Sousa, Sunita M. C.; Gill, Anthony J.; Serpell, Jonathan; Taubman, Kim; Christie, James; Carroll, Richard W.; Miller, Julie A.; Grossmann, Mathis	2021	Primary hyperparathyroidism in adults-(Part I) assessment and medical management: Position statement of the endocrine society of Australia, the Australian & New Zealand endocrine surgeons, and the Australian & New Zealand bone and mineral society	Clinical endocrinology	Review/Guideline
155	Mischis-Troussard, C; Goudet, P; Verges, B; Cougard, P; Tavernier, C; Mailletfert, JF	2000	Primary hyperparathyroidism with normal serum intact parathyroid hormone levels	QJM: monthly journal of the Association of Physicians	PROM not used
156	Misgar, Raiz Ahmad; Dar, Parvez Mohiuddin; Masoodi, Shariq Rashid; Ahmad, Munir; Wani, Khursheed Alam; Wani, Arshad Iqbal; Bashir, Mir Iftikhar	2016	Clinical and laboratory profile of primary hyperparathyroidism in Kashmir Valley: A single-center experience	Indian journal of endocrinology and metabolism	PROM not used
157	Mittendorf, Elizabeth A.; Wefel, Jeffrey S.; Meyers, Christina A.; Doherty, David; Shapiro, Suzanne E.; Lee, Jeffrey E.; Evans, Douglas B.; Perrier, Nancy D.	2007	Improvement of sleep disturbance and neurocognitive function after parathyroidectomy in patients with primary hyperparathyroidism	Endocrine practice : official journal of the American College of Endocrinology and the American Association of Clinical Endocrinologists	No measurement properties
158	Mohan, Bharth; Abuji, Kishore; Dahiya, Divya; Tandup, Cherring; Bhadada, Sanjay; Behera, Arunanshu	2021	Quality of life assessment after parathyroidectomy in symptomatic primary hyperparathyroidism using the SF-36 questionnaire	Turkish journal of surgery	No measurement properties
159	Montgomery, Kelsey B.; Fazendin, Jessica M.; Lindeman, Brenessa; Chen, Herbert	2021	Tired of Being Ignored: Fatigue as a Presenting Symptom in Primary Hyperparathyroidism	The Journal of surgical research	PROM not used

160	Morris, G.; Hearon, C. M.; Balachandran, D.; Bashura, L.; Wefel, J. S.; Dong, W.; Jackson, E.; Perrier, N. D.	2010	Prospective, randomized, controlled trial of parathyroidectomy versus observation in patients with â€œasymptomaticâ€ primary hyperparathyroidism	Journal of the American Geriatrics Society	Not primary research study
161	Morris, Lilah F.; Zelada, Juliette; Wu, Bian; Hahn, Theodore J.; Yeh, Michael W.	2010	Parathyroid surgery in the elderly	The oncologist	Review/Guideline
162	Murray, Sara E.; Pathak, Priya R.; Pontes, David S.; Schneider, David F.; Schaefer, Sarah C.; Chen, Herbert; Sippel, Rebecca S.	2013	Timing of symptom improvement after parathyroidectomy for primary hyperparathyroidism	Surgery	PROM not used
163	Murray, Sara E.; Pathak, Priya R.; Schaefer, Sarah C.; Chen, Herbert; Sippel, Rebecca S.	2014	Improvement of sleep disturbance and insomnia following parathyroidectomy for primary hyperparathyroidism	World journal of surgery	No measurement properties
164	Muthukrishnan, J.; Jha, Sangeeta; Modi, K. D.; Jha, R.; Kumar, J.; Verma, A.; Harikumar, K. V. S.; Patro, Kiran; Srinivas, B.; Kumaresan, K.; Ramasubba, Rayudu	2008	Symptomatic primary hyperparathyroidism: a retrospective analysis of fifty one cases from a single centre	The Journal of the Association of Physicians of India	PROM not used
165	Nct,	2016	Effect of Parathyroidectomy on Cardiovascular Health	https://clinicaltrials.gov/show/NCT02989428	Trial protocol
166	Nct,	2018	Gait and Balance Parameters Before and After Parathyroidectomy in Patients With Primary Hyperparathyroidism	https://clinicaltrials.gov/show/NCT03713671	Trial protocol
167	Nct,	2000	A Randomized Study of Surgery vs No Surgery in Patients With Mild Asymptomatic Primary Hyperparathyroidism	https://clinicaltrials.gov/show/NCT00004843	Trial protocol
168	Nct,	2009	A Study to Assess the Efficacy and Safety of Twice-Daily Dose Regimens of an Oral Calcimimetic Agent AMG 073 (Cinacalcet) in Primary Hyperparathyroidism (PHPT)	https://clinicaltrials.gov/show/NCT00936650	Trial protocol
169	Nct,	2009	Vitamin D Supplementation After Parathyroid Surgery	https://clinicaltrials.gov/show/NCT00982722	Trial protocol

170	Niederle, B.; Roka, R.; Woloszczuk, W.; Klaushofer, K.; Kovarik, J.; Schernthaner, G.	1987	Successful parathyroidectomy in primary hyperparathyroidism: a clinical follow-up study of 212 consecutive patients	Surgery	PROM not used
171	Nikitina, T. P.; Gladkova, I. N.; Rusakov, V. F.; Chernikov, R. A.; Karelina, Yu V.; Efremov, S. M.; Ionova, T. I.	2022	[Quality of life in patients with primary hyperparathyroidism after surgery]	Problemy endokrinologii	Not English
172	Norman, James; Lopez, Jose; Politz, Doug	2012	Cinacalcet (Sensipar) provides no measurable clinical benefits for patients with primary hyperparathyroidism and may accelerate bone loss with prolonged use	Annals of surgical oncology	PROM not used
173	Norman, James; Politz, Douglas; Lopez, Jose; Boone, Deva; Stojadinovic, Alexander	2015	Surgical cure of primary hyperparathyroidism ameliorates gastroesophageal reflux symptoms	World journal of surgery	No measurement properties
174	Nudelman, I.; Deutsch, A. A.; Reiss, R.	1983	Surgical treatment of primary hyperparathyroidism in the elderly patient	Israel journal of medical sciences	PROM not used
175	Numann, P. J.; Torppa, A. J.; Blumetti, A. E.	1984	Neuropsychologic deficits associated with primary hyperparathyroidism	Surgery	PROM not used
176	Ohrvall, U.; Akerstrom, G.; Ljunghall, S.; Lundgren, E.; Juhlin, C.; Rastad, J.	1994	Surgery for sporadic primary hyperparathyroidism in the elderly	World journal of surgery	PROM not used
177	Okamoto, T.; Gerstein, H. C.; Obara, T.	1997	Psychiatric symptoms, bone density and non-specific symptoms in patients with mild hypercalcemia due to primary hyperparathyroidism: a systematic overview of the literature	Endocrine journal	Review/Guideline
178	Okamoto, Takahiro; Kamo, Toshiko; Obara, Takao	2002	Outcome study of psychological distress and nonspecific symptoms in patients with mild primary hyperparathyroidism	Archives of surgery (Chicago, Ill. : 1960)	No measurement properties
179	Ozogul, B; Kisaoglu, A; Akcay, NM; Atamanalp, SS; Yildirgan, MI; Ozturk, G	2013	Clinical experience in the surgical treatment of primary hyperparathyroidism: 94 cases	Duzce Medical Journal	PROM not used
180	Pallan, Shelley; Khan, Aliya	2011	Primary hyperparathyroidism: Update on presentation, diagnosis, and management in primary care	Canadian family physician Medecin de famille canadien	Review/Guideline
181	Papavramidis, T. S.; Anagnostis, P.; Pliakos, I.; Tzikos, G.; Chorti, A.; Kotsa, K.; Michalopoulos, A.	2022	The impact of age on quality of life and frailty outcomes after parathyroidectomy in patients with primary hyperparathyroidism	Journal of endocrinological investigation	No measurement properties

182	Pappachan, Joseph M.; Lahart, Ian M.; Viswanath, Ananth K.; Borumandi, Farzad; Sodi, Ravinder; Metzendorf, Maria-Inti; Bongaerts, Brenda	2023	Parathyroidectomy for adults with primary hyperparathyroidism	The Cochrane database of systematic reviews	Review/Guideline
183	Parikh, Punam P.; Allan, Bassan J.; Lew, John I.	2014	Surgical treatment of patients with mildly elevated parathormone and calcium levels	World journal of surgery	PROM not used
184	Parks, K. A.; Parks, C. G.; Onwuameze, O. E.; Shrestha, S.	2017	Psychiatric complications of primary hyperparathyroidism and mild hypercalcemia	American Journal of Psychiatry	Not primary research study
185	Pasieka, Janice L.; Parsons, Louise; Jones, Jean	2009	The long-term benefit of parathyroidectomy in primary hyperparathyroidism: a 10-year prospective surgical outcome study	Surgery	No measurement properties
186	Patel, Neil; Mihai, Radu	2021	Assessing neuropsychological symptoms in primary hyperparathyroidism: Further work needed to confirm the findings	Surgery	Not primary research study
187	Pathak, Priya R.; Holden, Sara E.; Schaefer, Sarah C.; Leverson, Glen; Chen, Herbert; Sippel, Rebecca S.	2014	Elevated parathyroid hormone after parathyroidectomy delays symptom improvement	The Journal of surgical research	PROM not used
188	Peng, Zhen-Xing; Qin, Yong; Bai, Juan; Yin, Jin-Shu; Wei, Bo-Jun	2022	Analysis of the successful clinical treatment of 140 patients with parathyroid adenoma: A retrospective study	World journal of clinical cases	PROM not used
189	Pepe, Jessica; Badiali, Danilo; Biviano, Ivano; Nofroni, Italo; Romagnoli, Elisabetta; Cilli, Mirella; Piemonte, Sara; Cipriani, Cristiana; Colagenlo, Luciano; Minisola, Salvatore	2013	The effect of parathyroidectomy on chronic constipation in patients affected by primary hyperparathyroidism	Journal of Bone and Mineral Metabolism	PROM not used
190	Perez-Ruiz, L.; Lasheras-Alonso, M.; Gomez-Arbones, X.; Ros-Lopez, S.; Pelayo-Salas, A.; Salcedo-Mata, M. D.	2006	The effects of successful parathyroidectomy on clinical and biological manifestations of primary hyperparathyroidism	Acta chirurgica Belgica	PROM not used

191	Perrier, Nancy D.; Balachandran, Dave; Wefel, Jeffrey S.; Jimenez, Camilo; Busaidy, Naifa; Morris, George S.; Dong, Wenli; Jackson, Edward; Weaver, Storm; Gantela, Swaroop; Evans, Douglas B.; Grubbs, Elizabeth G.; Lee, Jeffrey E.	2009	Prospective, randomized, controlled trial of parathyroidectomy versus observation in patients with "asymptomatic" primary hyperparathyroidism	Surgery	No measurement properties
192	Perrier, Nancy D.; Coker, Laura H.; Rorie, Kashemi D.; Burbank, Nicole S.; Kirkland, Kimberly A.; Passmore, Leah V.; Tembreull, Terry; Stump, David A.; Laurienti, Paul J.	2006	Preliminary report: functional MRI of the brain may be the ideal tool for evaluating neuropsychologic and sleep complaints of patients with primary hyperparathyroidism	World journal of surgery	No measurement properties
193	Pfitzenmeyer, P.; Besancenot, J. F.; Verges, B.; Cougard, P.; Lorcerie, B.; Cercueil, J. P.; Monnier, V.; Turcu, A.; Gaudet, M.	1993	Primary hyperparathyroidism in very old patients	The European journal of medicine	PROM not used
194	Polistena, Andrea; Lucchini, Roberta; Monacelli, Massimo; Triola, Roberta; Avenia, Stefano; Barillaro, Ivan; Johnson, Louis Banka; Sanguinetti, Alessandro; Avenia, Nicola	2017	Current Indications for Surgical Treatment of Primary Hyperparathyroidism in the Elderly	The American surgeon	PROM not used
195	Politz, Douglas; Norman, James	2007	Hyperparathyroidism in patients over 80: clinical characteristics and their ability to undergo outpatient parathyroidectomy	Thyroid : official journal of the American Thyroid Association	PROM not used
196	Potts, J. T.	1991	NIH CONFERENCE - DIAGNOSIS AND MANAGEMENT OF ASYMPTOMATIC PRIMARY HYPERPARATHYROIDISM - CONSENUS DEVELOPMENT CONFERENCE STATEMENT	Annals of internal medicine	Review/Guideline
197	Pradeep, P. V.; Mishra, Anjali; Agarwal, Gaurav; Agarwal, Amit; Verma, A. K.; Mishra, S. K.	2008	Long-term outcome after parathyroidectomy in patients with advanced primary hyperparathyroidism and associated vitamin D deficiency	World journal of surgery	PROM not used

198	Prager, Gerhard; Kalaschek, Andreas; Kaczirek, Klaus; Passler, Christian; Scheuba, Christian; Sonneck, Gernot; Niederle, Bruno	2002	Parathyroidectomy improves concentration and retentiveness in patients with primary hyperparathyroidism	Surgery	PROM not used
199	Prasarttong-Osoth, Poramaporn; Wathanaoran, Pakpong; Imruetaicharoenchoke, Waraporn; Rojananin, Supakorn	2012	Primary hyperparathyroidism: 11-year experience in a single institute in Thailand	International journal of endocrinology	PROM not used
200	Pretorius, Mikkel; Lundstam, Karolina; Hellstrom, Mikael; Fagerland, Morten W.; Godang, Kristin; Mollerup, Charlotte; Fougner, Stine L.; Pernow, Ylva; Aas, Turid; Hessman, Ola; Rosen, Thord; Nordenstrom, Jorgen; Jansson, Svante; Heck, Ansgar; Bollerslev, Jens	2021	Effects of Parathyroidectomy on Quality of Life: 10 Years of Data From a Prospective Randomized Controlled Trial on Primary Hyperparathyroidism (the SIPH-Study)	Journal of bone and mineral research : the official journal of the American Society for Bone and Mineral Research	No measurement properties
201	Quiros, R. M.; Alef, M. J.; Wilhelm, S. M.; Djuricin, G.; Loviscek, K.; Prinz, R. A.	2003	Health-related quality of life in hyperparathyroidism measurably improves after parathyroidectomy	Surgery	No measurement properties
202	Raef, H.; Ingemansson, S.; Sobhi, S.; Sultan, A.; Ahmed, M.; Chaudhry, M.	2004	The effect of vitamin D status on the severity of bone disease and on the other features of primary hyperparathyroidism (pHPT) in a vitamin D deficient region	Journal of endocrinological investigation	PROM not used
203	Ramakant, P.; Verma, A. K.; Chand, G.; Mishra, A.; Agarwal, G.; Agarwal, A.; Mishra, S. K.	2011	Salutary effect of parathyroidectomy on neuropsychiatric symptoms in patients with primary hyperparathyroidism: evaluation using PAS and SF-36v2 scoring systems	Journal of postgraduate medicine	No measurement properties
204	Rao, D. Sudhaker; Phillips, Evelyn R.; Divine, George W.; Talpos, Gary B.	2004	Randomized controlled clinical trial of surgery versus no surgery in patients with mild asymptomatic primary hyperparathyroidism	The Journal of clinical endocrinology and metabolism	No measurement properties

205	Rasche, Renan Viola; Schuster, Frauke; Meurer, Natalie; Margariti, Theodora; Weyerbrock, Norbert; Rasche, Kurt; Dotzenrath, Cornelia	2021	Influence of Parathyroidectomy on Sleep Quality in Primary Hyperparathyroidism	Advances in experimental medicine and biology	No measurement properties
206	Rastad, J.; Joborn, C.; AkerstrÃ¶m, G.; Ljunghall, S.	1992	Incidence, type and severity of psychic symptoms in patients with sporadic primary hyperparathyroidism	J Endocrinol Invest	PROM not used
207	Reiher, Alexandra E.; Mazeh, Haggi; Schaefer, Sarah; Gould, Jon; Chen, Herbert; Sippel, Rebecca S.	2012	Symptoms of gastroesophageal reflux disease improve after parathyroidectomy	Surgery	No measurement properties
208	Repplinger, Daniel; Schaefer, Sarah; Chen, Herbert; Sippel, Rebecca S.	2009	Neurocognitive dysfunction: a predictor of parathyroid hyperplasia	Surgery	PROM not used
209	Rodgers, Steven E.; Lew, John I.; Solorzano, Carmen C.	2008	Primary hyperparathyroidism	Current opinion in oncology	Review/Guideline
210	Roka, R.; Niederle, B.; Kovarik, J.; Klaushofer, K.; Schernthaner, G.; Fritsch, A.	1987	Clinical long-term results after parathyroidectomy for primary hyperparathyroidism	Acta chirurgica Scandinavica	PROM not used
211	Rolighed, Lars; Amstrup, Anne Kristine; Jakobsen, Niels Frederik Breum; Sikjaer, Tanja; Mosekilde, Leif; Christiansen, Peer; Rejnmark, Lars	2014	Muscle function is impaired in patients with "asymptomatic" primary hyperparathyroidism	World journal of surgery	No measurement properties
212	Rolighed, Lars; Rejnmark, Lars; Sikjaer, Tanja; Heickendorff, Lene; Vestergaard, Peter; Mosekilde, Leif; Christiansen, Peer	2015	No beneficial effects of vitamin D supplementation on muscle function or quality of life in primary hyperparathyroidism: results from a randomized controlled trial	European journal of endocrinology	No measurement properties

213	Roman, Sanziana A.; Sosa, Julie Ann; Mayes, Linda; Desmond, Eric; Boudourakis, Leon; Lin, Rong; Snyder, Peter J.; Holt, Elizabeth; Udelsman, Robert	2005	Parathyroidectomy improves neurocognitive deficits in patients with primary hyperparathyroidism	Surgery	No measurement properties
214	Roman, Sanziana A.; Sosa, Julie Ann; Pietrzak, Robert H.; Snyder, Peter J.; Thomas, Daniel C.; Udelsman, Robert; Mayes, Linda	2011	The effects of serum calcium and parathyroid hormone changes on psychological and cognitive function in patients undergoing parathyroidectomy for primary hyperparathyroidism	Annals of surgery	No measurement properties
215	Roman, Sanziana; Sosa, Julie Ann	2007	Psychiatric and cognitive aspects of primary hyperparathyroidism	Current opinion in oncology	Review/Guideline
216	Ronni-Sivula, H.; Sivula, A.	1985	Long-term effect of surgical treatment on the symptoms of primary hyperparathyroidism	Annals of clinical research	PROM not used
217	Ruijs, C. D.; Ottow, R. T.; van Vroonhoven, T. J.	1994	Old age is not a contra-indication for surgery in patients with primary hyperparathyroidism	The Netherlands journal of medicine	PROM not used
218	Ryan, J. A., Jr.; Lee, F.	1997	Effectiveness and safety of 100 consecutive parathyroidectomies	American journal of surgery	PROM not used
219	Ryhanen, Eeva M.; Heiskanen, Ilkka; Sintonen, Harri; Valimaki, Matti J.; Roine, Risto P.; Schalin-Jantti, Camilla	2015	Health-related quality of life is impaired in primary hyperparathyroidism and significantly improves after surgery: a prospective study using the 15D instrument	Endocrine connections	No measurement properties
220	Schmidli, R. S.; Wilson, I.; Espiner, E. A.; Richards, A. M.; Donald, R. A.	1990	Aminopropylidine diphosphonate (APD) in mild primary hyperparathyroidism: effect on clinical status	Clinical endocrinology	PROM not used
221	Serdenes, Ryan; Lewis, Morgan; Chandrasekhara, Seetha	2021	A Clinical Review of the Psychiatric Sequelae of Primary Hyperparathyroidism	Cureus	Review/Guideline
222	Sevinc, B.; Damburaci, N.; Karahan, O.; Atasever, A.; Haciyanli, M.	2022	Evaluation of Swallowing Related Quality of Life and Health Related Quality of Life in Patients with Primary Hyperparathyroidism	INDIAN JOURNAL OF SURGERY	No measurement properties
223	Shah, Viral N.; Bhadada, Sanjay Kumar; Bhansali, Anil; Behera, A.; Bhattacharya, Anish; Nahar, Uma; Bhasin, Deepak; Vadera, Bhavin	2014	Effect of gender, biochemical parameters & parathyroid surgery on gastrointestinal manifestations of symptomatic primary hyperparathyroidism	The Indian journal of medical research	PROM not used

224	Shah-Becker, Shivani; Derr, Jonathan; Oberman, Benjamin S.; Baker, Aaron; Saunders, Brian; Carr, Michele M.; Goldenberg, David	2018	Early neurocognitive improvements following parathyroidectomy for primary hyperparathyroidism	The Laryngoscope	No measurement properties
225	Sheldon, David G.; Lee, Faye T.; Neil, Nancy J.; Ryan, John A., Jr.	2002	Surgical treatment of hyperparathyroidism improves health-related quality of life	Archives of surgery (Chicago, Ill. : 1960)	No measurement properties
226	Shinjo, S. K.; Pereira, R. M. R.; Borssatto, A. G. F.; Kochen, J. A. L.	2009	Musculoskeletal manifestations in primary hyperparathyroidism	Revista Brasileira de Reumatologia	Not English
227	Siddiqi, N. S.; Najafi, B.; Olsen, C.; Castro, M.; Suliburk, J. W.	2017	Objective Assessment of Functional and Motor-Cognitive Outcomes among Asymptomatic Primary Hyperparathyroidism Patients Undergoing Parathyroidectomy Using Wearable Technologies: A Pilot Study Toward Better Informed Clinical Decision-Making	Journal of the American College of Surgeons	Not primary research study
228	Silverberg, Shonni J.	2002	Non-classical target organs in primary hyperparathyroidism	Journal of bone and mineral research : the official journal of the American Society for Bone and Mineral Research	Review/Guideline
229	Silverberg, Shonni J.; Bilezikian, John P.	2006	The diagnosis and management of asymptomatic primary hyperparathyroidism	Nature clinical practice. Endocrinology & metabolism	Review/Guideline
230	Silverberg, Shonni J.; Clarke, Bart L.; Peacock, Munro; Bandeira, Francisco; Boutroy, Stephanie; Cusano, Natalie E.; Dempster, David; Lewiecki, E. Michael; Liu, Jian-Min; Minisola, Salvatore; Rejnmark, Lars; Silva, Barbara C.; Walker, Marcella D.; Bilezikian, John P.	2014	Current issues in the presentation of asymptomatic primary hyperparathyroidism: proceedings of the Fourth International Workshop	The Journal of clinical endocrinology and metabolism	Review/Guideline

231	Silverberg, Shonni J.; Lewiecki, E. Michael; Mosekilde, Leif; Peacock, Munro; Rubin, Mishaela R.	2009	Presentation of asymptomatic primary hyperparathyroidism: proceedings of the third international workshop	The Journal of clinical endocrinology and metabolism	Review/Guideline
232	Silverberg, Shonni J.; Walker, Marcella D.; Bilezikian, John P.	2013	Asymptomatic primary hyperparathyroidism	Journal of clinical densitometry : the official journal of the International Society for Clinical Densitometry	Review/Guideline
233	Singh Ospina, N.; Maraka, S.; Rodriguez-Gutierrez, R.; Espinosa de Ycaza, A. E.; Jasim, S.; Gionfriddo, M.; Castaneda-Guarderas, A.; Brito, J. P.; Al Nofal, A.; Erwin, P.; Wermers, R.; Montori, V.	2016	Comparative efficacy of parathyroidectomy and active surveillance in patients with mild primary hyperparathyroidism: a systematic review and meta-analysis	Osteoporosis international : a journal established as result of cooperation between the European Foundation for Osteoporosis and the National Osteoporosis Foundation of the USA	Review/Guideline
234	Sivula, A.; Ronni-Sivula, H.	1984	The changing picture of primary hyperparathyroidism in the years 1956-1979	Annales chirurgiae et gynaecologiae	PROM not used
235	Slitt, Gavin T.; Lavery, Hugh; Morgan, Anthony; Bernstein, Bruce; Slavin, James; Karimeddini, Mozaferiddin K.; Kozol, Robert A.	2005	Hyperparathyroidism but a negative sestamibi scan: a clinical dilemma	American journal of surgery	No measurement properties
236	Solomon, B. L.; Schaaf, M.; Smallridge, R. C.	1994	Psychologic symptoms before and after parathyroid surgery	The American journal of medicine	No measurement properties
237	Somuncu, Erkan; Kara, Yasin	2021	The effect of parathyroidectomy on quality of life in primary hyperparathyroidism: evaluation with using sf-36 and phpqol questionnaire	Endocrine journal	No measurement properties
238	Starker, Lee F.; Bjorklund, Peyman; Theoharis, Constantine; Long, William D., 3rd; Carling, Tobias; Udelsman, Robert	2011	Clinical and histopathological characteristics of hyperparathyroidism-induced hypercalcemic crisis	World journal of surgery	PROM not used

239	Stechman, Michael J.; Weisters, Mary; Gleeson, Fergus V.; Sadler, Gregory P.; Mihai, Radu	2009	Parathyroidectomy is safe and improves symptoms in elderly patients with primary hyperparathyroidism (PHPT)	Clinical endocrinology	No measurement properties
240	Stephen, Antonia E.; Mannstadt, Michael; Hodin, Richard A.	2017	Indications for Surgical Management of Hyperparathyroidism: A Review	JAMA surgery	Review/Guideline
241	Steward, David L.; Bhatki, Amol M.; Falciglia, Mercedes	2008	The Effects of Surgery for Primary Hyperparathyroidism	Otolaryngology-Head & Neck Surgery	Review/Guideline
242	Storvall, Sara; Ryhanen, Eeva M.; Heiskanen, Ilkka; Sintonen, Harri; Roine, Risto P.; Schalin-Jantti, Camilla	2017	Surgery Significantly Improves Neurocognition, Sleep, and Blood Pressure in Primary Hyperparathyroidism: A 3-Year Prospective Follow-Up Study	Hormone and metabolic research = Hormon- und Stoffwechselforschung = Hormones et metabolisme	No measurement properties
243	Sun, B.; Guo, B.; Wu, B.; Kang, J.; Deng, X.; Zhang, Z.; Fan, Y.	2018	Characteristics, management, and outcome of primary hyperparathyroidism at a single clinical center from 2005 to 2016	Osteoporosis international : a journal established as result of cooperation between the European Foundation for Osteoporosis and the National Osteoporosis Foundation of the USA	PROM not used
244	Sywak, Mark S.; Knowlton, Sarah T.; Pasieka, Janice L.; Parsons, Louise L.; Jones, Jean	2002	Do the National Institutes of Health consensus guidelines for parathyroidectomy predict symptom severity and surgical outcome in patients with primary hyperparathyroidism?	Surgery	No measurement properties
245	Szalat, Auryan; Tamir, Noa; Mazeh, Haggi; Newman, J. P.	2022	Successful parathyroidectomy improves cognition in patients with primary hyperparathyroidism: A prospective study in a tertiary medical center and comprehensive review of the literature	Frontiers in endocrinology	No measurement properties
246	Talpos, G. B.; Bone, H. G., 3rd; Kleerekoper, M.; Phillips, E. R.; Alam, M.; Honasoge, M.; Divine, G. W.; Rao, D. S.	2000	Randomized trial of parathyroidectomy in mild asymptomatic primary hyperparathyroidism: patient description and effects on the SF-36 health survey	Surgery	No measurement properties

247	Tezelman, S.; Rodriguez, J. M.; Shen, W.; Siperstein, A. E.; Duh, Q. Y.; Clark, O. H.	1995	Primary hyperparathyroidism in patients who have received radiation therapy and in patients who have not received radiation therapy	Journal of the American College of Surgeons	PROM not used
248	Tezelman, S.; Shen, W.; Shaver, J. K.; Siperstein, A. E.; Duh, Q. Y.; Klein, H.; Clark, O. H.	1993	Double parathyroid adenomas. Clinical and biochemical characteristics before and after parathyroidectomy	Annals of surgery	PROM not used
249	Trofimiuk, M.; Pach, D.; Hubalewska-Dydejczyk, A.	2010	Asymptomatic primary hyperparathyroidism: Management and implications	Recent Patents on Endocrine, Metabolic and Immune Drug Discovery	Review/Guideline
250	Trombetti, A.; Christ, E. R.; Henzen, C.; Gold, G.; Brandle, M.; Herrmann, F. R.; Torriani, C.; Triponez, F.; Kraenzlin, M.; Rizzoli, R.; Meier, C.	2016	Clinical presentation and management of patients with primary hyperparathyroidism of the Swiss Primary Hyperparathyroidism Cohort: a focus on neuro-behavioral and cognitive symptoms	Journal of endocrinological investigation	No measurement properties
251	Tsukahara, Kiyoaki; Sugitani, Iwao; Fujimoto, Yoshihide; Kawabata, Kazuyoshi	2008	Surgery did not improve the subjective neuropsychological symptoms of patients with incidentally detected mild primary hyperparathyroidism	European archives of oto-rhino-laryngology : official journal of the European Federation of Oto-Rhino-Laryngological Societies (EUFOS) : affiliated with the German Society for Oto-Rhino-Laryngology - Head and Neck Surgery	PROM not used
252	Turken, S. A.; Cafferty, M.; Silverberg, S. J.; De La Cruz, L.; Cimino, C.; Lange, D. J.; Lovelace, R. E.; Bilezikian, J. P.	1989	Neuromuscular involvement in mild, asymptomatic primary hyperparathyroidism	Am J Med	PROM not used
253	Turner, J.	2009	Hypercalcaemia and primary hyperparathyroidism	Medicine	Review/Guideline
254	Turner, J. J. O.	2017	Hypercalcaemia and primary hyperparathyroidism	Medicine (United Kingdom)	Review/Guideline

255	Tzikos, Georgios; Chorti, Angeliki; Evangelos, Sykaras; Boura, Evangelia; Manani, Christina; Adamidou, Fotini; Tziatzios, Ioannis; Zisi, Anna; Economou, Fotios; Toulis, Konstantinos; Pliakos, Ioannis; Michalopoulos, Antonios; Papavramidisos, Theodosios	2021	Quality of Life in Patients With Asymptomatic Primary Hyperparathyroidism After Parathyroidectomy: A 3-Year Longitudinal Study	Endocrine practice : official journal of the American College of Endocrinology and the American Association of Clinical Endocrinologists	No measurement properties
256	Uden, P.; Chan, A.; Duh, Q. Y.; Siperstein, A.; Clark, O. H.	1992	Primary hyperparathyroidism in younger and older patients: symptoms and outcome of surgery	World journal of surgery	PROM not used
257	Vadhwana, B.; Groot-Wassink, T.	2018	Impact on quality of life after parathyroidectomy for primary hyperparathyroidism	British Journal of Surgery	Not primary research study
258	Vadhwana, Bhamini; Currow, Chelise; Bowers, David; Groot-Wassink, Thomas	2021	Impact on Quality of Life After Parathyroidectomy for Asymptomatic Primary Hyperparathyroidism	The Journal of surgical research	No measurement properties
259	Valle Diaz de la Guardia, Francisco; Arrabal Martin, Miguel; Arrabal Polo, Miguel Angel; Quirosa Flores, Susana; Mijan Ortiz, Jose Luis; Zuluaga Gomez, Armando	2010	Renal lithiasis in patients with primary hyperparathyroidism. Evolution and treatment	Archivos espanoles de urologia	Not English
260	Venkat, Raghunandan; Kouniavsky, Guennadi; Tufano, Ralph P.; Schneider, Eric B.; Dackiw, Alan P. B.; Zeiger, Martha A.	2012	Long-term outcome in patients with primary hyperparathyroidism who underwent minimally invasive parathyroidectomy	World journal of surgery	No measurement properties
261	Vera, Lara; Accornero, Martina; Dolcino, Mara; Oddo, Silvia; Giusti, Massimo	2014	Five-year longitudinal evaluation of mild primary hyperparathyroidism - medical treatment versus clinical observation	Endokrynologia Polska	No measurement properties
262	Veras, Andreia; Maia, Juliana; Mesquita, Patricia; Eufrazino, Catia; Bandeira, Francisco	2013	Lower quality of life in longstanding mild primary hyperparathyroidism	Arq Bras Endocrinol Metabol	No measurement properties

263	Vestergaard, Peter; Mosekilde, Leif	2003	Cohort study on effects of parathyroid surgery on multiple outcomes in primary hyperparathyroidism	BMJ (Clinical research ed.)	PROM not used
264	Voss, Lara; NÃ³brega, Maira; Bandeira, Leonardo; Griz, Luiz; Rocha-Filho, Pedro Augusto Sampaio; Bandeira, Francisco	2020	Impaired physical function and evaluation of quality of life in normocalcemic and hypercalcemic primary hyperparathyroidism	Bone	No measurement properties
265	Walgenbach, S.; Hommel, G.; Bernhard, G.; Junginger, T.	2000	[Surgical therapy of primary hyperparathyroidism. Quality of life after 10 years prospective observation]	Operative Therapie des primaren Hyperparathyreoidismus. Lebensqualitat nach 10jahriger prospektiver Beobachtung.	Not English
266	Walker, Marcella D.; McMahon, Donald J.; Inabnet, William B.; Lazar, Ronald M.; Brown, Ijeoma; Vardy, Susan; Cosman, Felicia; Silverberg, Shonni J.	2009	Neuropsychological features in primary hyperparathyroidism: a prospective study	The Journal of clinical endocrinology and metabolism	No measurement properties
267	Walker, Marcella D.; Silverberg, Shonni J.	2007	Parathyroidectomy in asymptomatic primary hyperparathyroidism: improves "bones" but not "psychic moans"	The Journal of clinical endocrinology and metabolism	Not primary research study
268	Walker, Marcella Donovan; Rubin, Mishaela; Silverberg, Shonni J.	2013	Nontraditional manifestations of primary hyperparathyroidism	Journal of clinical densitometry : the official journal of the International Society for Clinical Densitometry	Review/Guideline
269	Walker, Marcella Donovan; Silverberg, Shonni J.	2021	Quality of Life in Primary Hyperparathyroidism Revisited: Keep Calm and Carry on?	Journal of bone and mineral research : the official journal of the American Society for Bone and Mineral Research	Not primary research study
270	Walker, Marcella; Silverberg, Shonni J.	2021	Nontraditional Aspects of Sporadic Primary Hyperparathyroidism	Endocrinology and metabolism clinics of North America	Review/Guideline

271	Walker, Regina Paloyan; Paloyan, Edward; Gopalsami, Chellam	2004	Symptoms in patients with primary hyperparathyroidism: muscle weakness or sleepiness	Endocrine practice : official journal of the American College of Endocrinology and the American Association of Clinical Endocrinologists	No measurement properties
272	Wang, G.; Jin, Z.; Wang, F.; Li, T.	2022	Diagnosis and surgical treatment of primary hyperparathyroidism in the elderly people	Chinese Journal of Endocrine Surgery	Not English
273	Wang, Yuting; Xin, Yunhui; Zhao, Teng; Shen, Hong; Liu, Xing; Wang, Jiacheng; Wang, Qian; Shen, Rongfang; Feng, Dalin; Wei, Bojun	2023	PTH levels, sleep quality, and cognitive function in primary hyperparathyroidism	Endocrine	No measurement properties
274	Weber, Theresia; Eberle, Julia; Messelhauser, Ursula; Schiffmann, Leif; Nies, Christoph; Schabram, Jochen; Zielke, Andreas; Holzer, Katharina; Rottler, Edit; Henne-Bruns, Doris; Keller, Monika; von Wietersheim, Jorn	2013	Parathyroidectomy, elevated depression scores, and suicidal ideation in patients with primary hyperparathyroidism: results of a prospective multicenter study	JAMA surgery	No measurement properties
275	Weber, Theresia; Keller, Monika; Hense, Isabella; Pietsch, Alexander; Hinz, Ulf; Schilling, Tobias; Nawroth, Peter; Klar, Ernst; Buchler, Markus W.	2007	Effect of parathyroidectomy on quality of life and neuropsychological symptoms in primary hyperparathyroidism	World journal of surgery	No measurement properties
276	Wells, S. A., Jr.	1991	Surgical therapy of patients with primary hyperparathyroidism: long-term benefits	Journal of bone and mineral research : the official journal of the American Society for Bone and Mineral Research	Review/Guideline
277	Wilhelm, Scott M.; Lee, John; Prinz, Richard A.	2004	Major depression due to primary hyperparathyroidism: a frequent and correctable disorder	The American surgeon	PROM not used

278	Wilhelm, Scott M.; Wang, Tracy S.; Ruan, Daniel T.; Lee, James A.; Asa, Sylvia L.; Duh, Quan-Yang; Doherty, Gerard M.; Herrera, Miguel F.; Pasieka, Janice L.; Perrier, Nancy D.; Silverberg, Shonni J.; Solorzano, Carmen C.; Sturgeon, Cord; Tublin, Mitchell E.; Udelsman, Robert; Carty, Sally E.	2016	The American Association of Endocrine Surgeons Guidelines for Definitive Management of Primary Hyperparathyroidism	JAMA surgery	Review/Guideline
279	Wu, James X.; Yeh, Michael W.	2016	Asymptomatic Primary Hyperparathyroidism: Diagnostic Pitfalls and Surgical Intervention	Surgical oncology clinics of North America	Review/Guideline
280	YÄ±lmaz, Banu; Toruner, Fusun; Konca, Ceyla; Turhan Iyidir, Ozlem; Kaya, Burhaneddin; Ã‡akÄ±r, Nuri	2017	Neuropsychological Changes and Health-related Quality of Life in Patients with Asymptomatic Primary Hyperparathyroidism	Turkish Journal of Endocrinology and Metabolism	No measurement properties
281	Yadav, Sanjay Kumar; Mishra, Saroj Kanta; Mishra, Anjali; Mayilvagnan, Sabaretnam; Chand, Gyan; Agarwal, Gaurav; Agarwal, Amit; Verma, Ashok Kumar	2018	Changing Profile of Primary Hyperparathyroidism Over Two and Half Decades: A Study in Tertiary Referral Center of North India	World J Surg	PROM not used
282	Ye, Zhikang; Silverberg, Shonni J.; Sreekanta, Ashwini; Tong, Kyle; Wang, Ying; Chang, Yaping; Zhang, Mengmeng; Guyatt, Gordon; Tangamornsuksun, Wimonchat; Zhang, Yi; Manja, Veena; Bakaa, Layla; Couban, Rachel J.; Brandi, Maria Luisa; Clarke, Bart; Khan, Aliya A.; Mannstadt, Michael; Bilezikian, John P.	2022	The Efficacy and Safety of Medical and Surgical Therapy in Patients With Primary Hyperparathyroidism: A Systematic Review and Meta-Analysis of Randomized Controlled Trials	Journal of bone and mineral research : the official journal of the American Society for Bone and Mineral Research	Review/Guideline

283	Yilmaz, Banu; Toruner, Fusun; Konca, Ceyla; Turham Iyidir, Ozlem; Kaya, Burhaneddin; Cakir, Nuri	2017	Neuropsychological Changes and Health-related Quality of Life in Patients with Asymptomatic Primary Hyperparathyroidism	Turkish Journal of Endocrinology and Metabolism	No measurement properties
284	Yoo, Jenny Y.; Yip, Linwah; Armstrong, Michaele J.; Carty, Sally E.; Kelley, Meghan L.; Stang, Michael T.; McCoy, Kelly L.	2016	Does impotence improve after parathyroidectomy in men with primary hyperparathyroidism?	Surgery	PROM not used
285	Zanocco, Kyle; Butt, Zeeshan; Kaltman, David; Elaraj, Dina; Cella, David; Holl, Jane L.; Sturgeon, Cord	2015	Improvement in patient-reported physical and mental health after parathyroidectomy for primary hyperparathyroidism	Surgery	No measurement properties
286	Zarnegar, R.; Clark, O. H.	2007	Current indications and decision-making leading to parathyroidectomy: A surgical viewpoint	Clinical Reviews in Bone and Mineral Metabolism	Review/Guideline
287	Zhang, L. Y.; Chen, Y.; Ao, Y. Z.	2023	Potential indicators for hyperparathyroidism progression: Calcium, phosphorus, alkaline phosphatase, 25 hydroxyvitamin D and hemoglobin	TROPICAL JOURNAL OF PHARMACEUTICAL RESEARCH	No measurement properties
288	Zhu, Catherine Y.; Nguyen, Dalena T.; Yeh, Michael W.	2019	Who Benefits from Treatment of Primary Hyperparathyroidism?	The Surgical clinics of North America	Review/Guideline
289		2007	Parathyroidectomy improves BMD and quality-of-life in patients with asymptomatic PHPT	Nature Clinical Practice Endocrinology and Metabolism	Not primary research study
