# Supplementary material for: Patient-reported outcome measures for primary hyperparathyroidism: a systematic review of measurement properties
Source: Health Qual Life Outcomes. 2024 Apr 2;22:31. doi: 10.1186/s12955-024-02248-9 (PMC10988805; doi:10.1186/s12955-024-02248-9)
Supplement: Supplementary file 6 — Supplementary Material 6 [file 12955_2024_2248_MOESM6_ESM.docx]

Additional File 3. Results of studies on measurement properties for the SF-36.

| **PROM (ref)** | **Country (language) in which the PROM was evaluated** | **Structural validity** | | | **Internal consistency** | | | | **Cross-cultural validity\measurement invariance** | | | | | **Reliability** | | |
| --- | --- | --- | --- | --- | --- | --- | --- | --- | --- | --- | --- | --- | --- | --- | --- | --- |
|  |  | n | Meth qual | Result (rating) | n | Meth qual | | Result (rating) | n | Meth qual | | Result (rating) | | n | Meth qual | Result (rating) |
| SF-36 (Burney 1996) | USA (English) |  |  |  | 59 | Doubtful* | | Cronbach’s alpha 0.83-0.93 (+) |  |  | |  | |  |  |  |
| SF-36 (Burney 1998) | USA (English) |  |  |  | 140 | Doubtful* | | Cronbach’s alpha 0.83-0.86 (+) |  |  | |  | |  |  |  |
| SF-36 (Burney 1999) | USA (English) |  |  |  | 155 | Doubtful* | | Cronbach’s alpha 0.83-0.86 (+) |  |  | |  | |  |  |  |
| **Pooled or summary result (overall rating)** | |  |  |  | **354** |  | | **(?)†** |  |  | |  | |  |  |  |
| **PROM** | **Country (language) in which the PROM was evaluated** | **Measurement error** | | | **Criterion validity** | | | **Hypotheses testing** | | | | | **Responsiveness** | | | |
|  |  | n | Meth qual | Result (rating) | n | Meth qual | Result (rating) | n | Meth qual | | Result (rating) | | n | | Meth qual | Result (rating) |
| SF-36 (Burney 1996) |  |  |  |  |  |  |  |  |  | |  | |  | |  |  |
| SF-36 (Burney 1998) |  |  |  |  |  |  |  |  |  | |  | |  | |  |  |
| SF-36 (Burney 1999) |  |  |  |  |  |  |  |  |  | |  | |  | |  |  |
| **Pooled or summary result (overall rating)** | |  |  |  |  |  |  |  |  | |  | |  | |  |  |

* Rated Doubtful as per standard 1 for internal consistency: it is unclear whether the scale or subscale is unidimensional as no information is available on structural validity.

† Rated indeterminate against the Updated Criteria for Good Measurement Properties because no studies on structural validity were available.
